# Supplementary material for: Introspective access to value-based multi-attribute choice processes
Source: Nat Commun. 2025 Apr 20;16:3733. doi: 10.1038/s41467-025-59080-y (PMC12009298; doi:10.1038/s41467-025-59080-y)
Supplement: Supplementary file 1 — Supplementary Information [file 41467_2025_59080_MOESM1_ESM.pdf]

Introspective access to value-based multi-attribute choice processes: Supplementary Information

Adam Morris[1*], Ryan W. Carlson[2], Hedy Kober[3†], and Molly J. Crockett[1*†]

[1]Department of Psychology, Princeton University, Princeton, NJ, USA
[2]Booth School of Business, University of Chicago, Chicago, IL, USA
[3]Department of Psychology, University of California: Berkeley, Berkeley, CA, USA

*Corresponding authors. Correspondence should be addressed to:
am9578@princeton.edu or mj.crockett@princeton.edu

†Joint supervisors.

All data, code, and study materials can be found at
https://doi.org/10.17605/OSF.IO/TMQJU.

Table of Contents:

Supplementary methods 3
1. Study procedures 3
1.1. Option attributes 3
1.2. Exact text of questions in Part 2 5
1.3. Additional questions and individual-difference battery 17
1.4. Self-reported method of answering self-report questions 21
1.5. How does the ACP task differ from prior work on awareness in multi-attribute choice? 22
1.6. Details of Study 4 (expert prediction study) 23
2. Analysis 26
2.1. Modeling procedure 27
2.2. Computing the probability of each heuristic 30
2.3. Simulation procedure 31
2.4. Regression analyses 32
2.5. Determining chance levels of accuracy measures 32
Supplementary discussion 33
3. Supplementary results for Studies 1-2 33
3.1. Participants 33
3.2. Quality control checks 34
3.3. Plots for Study 1B 36
3.4. Descriptives of which heuristics & weights people used and reported using 37
3.5. Analyzing method accuracy for each heuristic separately 40
3.6. Correlation between weight and method accuracy 41
3.7. Confounders 41
3.8. Individual difference measures 42
3.9. Alternative approaches to analyzing attribute weights 46
3.10. Comparison of weight accuracy results to those reported in past work 48
4. Supplementary results for Studies 3a-3b 48
4.1. Are observers more accurate when they are demographically similar to their paired decider? 49
4.2. Variance decomposition of decider vs. observer reports 50
5. Supplementary results for Study 4 51
Supplementary references 53

Supplementary methods

1. Study procedures

1.1. Option attributes

In the home variant, each home was characterized by: size of home (which ranged from 600 to 1800 sq. ft.); size of yard (which ranged from 0 to 600 square feet); quality of kitchen, quality of heat/AC system, and reputation of closest school (which all ranged on a five point scale from “Very Bad” to “Very Good”); amount of traffic (which ranged on a four point scale from “Very Little” to “A Lot”); noise pollution (which ranged on a five point scale from “Very Low” to “Very High”); year building was built (which ranged from 1950 to 2020); and closeness to parks (which ranged from 10 to 30 minutes away). Here is an example choice trial from the home variant:

	**Home A**	**Home B**
**Amount of traffic**	A Little	A Lot
**Year building was built**	1965	1986
**Size of yard**	400 square feet	50 square feet
**Quality of heat/AC system**	Very Bad	Very Good
**Reputation of closest school**	Moderate	Very Good
**Quality of kitchen**	Moderate	Very Good
**Closeness to parks**	15 min away	30 min away
**Size of home**	1800 square feet	600 square feet
**Noise pollution**	Very High	Very Low

In Study 1A, the set of home options used in choice trials was pre-determined; we chose a set that, based on pilot data, we believed would produce a sufficient spread of

preferences and choices across participants. In Study 1B, the choice trials were randomly generated for each participant; we ensured that, for each randomly generated pair of options, each attribute differed between the two options by at least two steps on the scale.

In the movie variant, each movie was characterized by: quality of humor, plot, creativity, action scenes, romantic scenes, soundtrack, visuals, dialogue, and acting (all ranging on a five point scale from “Very Bad” to “Very Good”). Each option in the movie variant was drawn from the MovieLens database, which uses machine learning to aggregate user ratings of movies across many dimensions[1–3]. We chose pairs of movies such that (a) in each pair, the two movies differed from each other on most dimensions, and (b) the attributes were minimally correlated with each other across the whole set of movies (most correlations between attribute pairs were between 0 - 0.2, with none exceeding 0.4).

Here is an example choice trial from the movie variant:

	**Movie A**	**Movie B**
**Humor**	Very Bad	Bad
**Plot**	Bad	Very Bad
**Creativity**	Very Bad	Moderate
**Action Scenes**	Bad	Very Bad
**Romantic Scenes**	Very Bad	Very Good
**Soundtrack**	Very Bad	Moderate
**Visuals**	Moderate	Very Bad
**Dialogue**	Very Good	Very Bad
**Acting**	Good	Bad

1.2. Exact text of questions in Part 2

Here we include the exact text of the key self-report questions in Part 2 of the ACP task. We only include the text from Study 1A; the rest can be found on our OSF page.

Here is the text for the attribute weight questions. Note that the order of the three types of weight questions (graded, binary, single-attribute) was randomized for each participant.

Introductory instructions:

In this section, we want to know **how much you cared about each feature of the homes when making your decisions.**

For instance, we'll ask you how much you cared about the homes' closeness to parks when making your decisions, how much you cared about the reputation of the closest school, and so on.

< Previous   Next >

We'll ask you a bunch of different questions about this. Some of the questions might not accurately describe how you made your decisions. Other times, the questions might feel redundant or repetitive.

That's okay -- please **just do your best to answer each question as accurately as possible.**

< Previous   Next >

Also, we specifically want to know how much you cared about each feature **while making your decisions in Section 1**, NOT how much you care about each feature in real life.

For instance, imagine that, in real life, you care a lot about the amount of traffic near your home -- but in this task, you didn't consider the amount of traffic at all in your choices. In that case, you would **say that you didn't care about the amount of traffic at all**.

< Previous   Next >

Binary weight questions:

First, we'll ask **which** features of the homes you cared about when making your decisions. This is what the question will look like:

Which features did you care about when making your choices?

(You can select as little as just 1 feature if you only cared about 1, or as many as all 9 features if you cared about all of them.)

- ☐ Closeness to parks
- ☐ Noise pollution
- ☐ Reputation of closest school
- ☐ Year building was built
- ☐ Size of yard
- ☐ Quality of kitchen
- ☐ Quality of heat/AC system
- ☐ Amount of traffic
- ☐ Size of home

So if you cared about the amount of noise pollution and the amount of traffic, and didn't care about any of the other features, you'd check 'Amount of noise pollution' and 'Amount of traffic' and not check any of the others.

When you're ready, press 'Next'.

< Previous | Next >

Which features did you care about when making your choices?

(You can select as little as just 1 feature if you only cared about 1, or as many as all 9 features if you cared about all of them.)

- ☐ Closeness to parks
- ☐ Noise pollution
- ☐ Reputation of closest school
- ☐ Year building was built
- ☐ Size of yard
- ☐ Quality of kitchen
- ☐ Quality of heat/AC system
- ☐ Amount of traffic
- ☐ Size of home

Continue

Single attribute questions:

Second, we'll ask **which single feature of the homes** you <u>MOST</u> used to make your decisions. The question will look like this:

What single feature did you MOST use to make your choices?

- ○ Closeness to parks
- ○ Noise pollution
- ○ Reputation of closest school
- ○ Year building was built
- ○ Size of yard
- ○ Quality of kitchen
- ○ Quality of heat/AC system
- ○ Amount of traffic
- ○ Size of home

For instance, maybe you primarily made your decision based on the homes' closeness to parks. If so, you would select 'Closeness to parks' for this question.

When you're ready, press 'Next'.

< Previous | Next >

What single feature did you MOST use to make your choices?

- ○ Closeness to parks
- ○ Noise pollution
- ○ Reputation of closest school
- ○ Year building was built
- ○ Size of yard
- ○ Quality of kitchen
- ○ Quality of heat/AC system
- ○ Amount of traffic
- ○ Size of home

Continue

Graded weight questions (we show only one example):

Third, we'll ask you **how much** you cared about each feature when making your decisions. Here's what the questions will look like:

Size of home

0 1 2 3 4 5 6 7 8

Didn't care about it at all | Cared a little | Cared somewhat | Cared a lot | It completely determined my choices

How much did you care about this feature when making your decisions?

So if you didn't care about the size of the home at all, you'd move the slider to 0; if you cared a moderate amount, you'd move the slider to around 4; and if it completely determined your choice, you'd move the slider to 8. And you can move the slider anywhere in between.

When you're ready, press 'Next'.

< Previous Next >

Quality of heat/AC system

0 1 2 3 4 5 6 7 8

Didn't care about it at all | Cared a little | Cared somewhat | Cared a lot | It completely determined my choices

How much did you care about this feature when making your decisions?

Here is the text for the heuristics questions. Note that the order of the three heuristics was randomized.

Introductory instructions:

On the next few pages, we're going to ask you some questions about the **big-picture decision-making strategies** people might use when choosing between homes in Section 1.

The strategies are not mutually exclusive, or all-or-nothing; people could be somewhere in the middle, or use one strategy on some trials and then another strategy on other trials. The strategies are meant to capture aspects of how people made their choices, not necessarily to describe their entire thought process. Just do your best to understand each strategy and answer the questions as accurately as you can.

< Previous Next >

Single attribute heuristic:

First, we're going to describe two strategies -- **Strategy A** and **Strategy B** -- that a person could have used to make choices in Section 1.

< Previous Next >

Strategies A and **B** are about the number of features of the homes that a person cared about when making their choices.

< Previous Next >

A person using **Strategy A** made all their decisions based off a **single, all-important feature** and did not consider the other features.

For instance, if someone only cared about the size of the home (and no other features) when making all their decisions, their strategy might look like this:

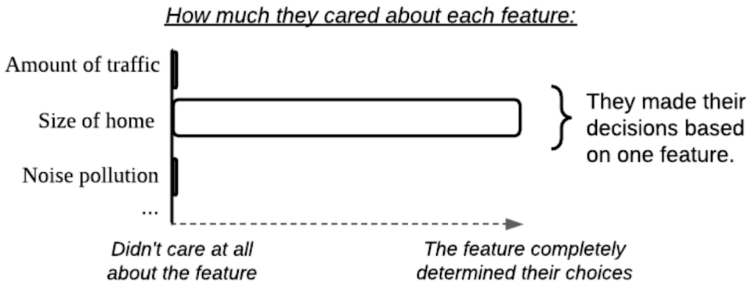

Of course, the person might have cared about a different feature than the one in this example. But the point is that they only cared about one feature throughout all their choices.

< Previous | Next >

In contrast, a person using **Strategy B** considered **multiple features** of the homes when making each of their decisions.

For instance, if someone cared about the amount of traffic, the size of the home, and the noise pollution when making each of their decisions, their strategy might look like this:

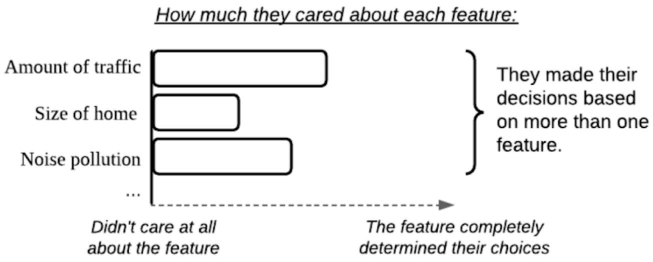

Of course, the person might have cared about different features than the ones in this example. But the point is that, during each choice, they cared about more than one feature.

< Previous | Next >

Here are the two strategies described again, for reference.

**Strategy A:** The person made all their decisions based off a **single, all-important feature** and did not consider the other features.	**Strategy B:** The person considered **multiple features** of the homes when making each of their choices.

When you feel like you've understood the two strategies, press 'Continue'.

Continue

Now, read the following description of a person, and answer: **Which of the two strategies better reflects how this person made their choices?**

Person description: When making his choices between homes in Section 1, Ricardo only cared about the amount of traffic; he always just chose whichever home had less traffic.

**Strategy A much better** describes how this person made their choices	**Strategy A somewhat better** describes how this person made their choices	**Both strategies equally** describe how this person made their choices	**Strategy B somewhat better** describes how this person made their choices	**Strategy B much better** describes how this person made their choices

Continue

Now, read this description of a different person, and answer again: **Which of the two strategies better reflects how this person made their choices?**

Person description: When making her choices between homes in Section 1, Sarah cared about three features: closeness to parks, reputation of the closest school, and the quality of the heat/AC system. She cared about the school reputation the most, but also cared a little about the closeness to parks and the heat/AC quality.

**Strategy A much better** describes how this person made their choices	**Strategy A somewhat better** describes how this person made their choices	**Both strategies equally** describe how this person made their choices	**Strategy B somewhat better** describes how this person made their choices	**Strategy B much better** describes how this person made their choices

Continue

Next, we want to know: Which of the two strategies better reflects how **YOU** made **YOUR** choices in Section 1 of the task?

**Strategy A much better** describes how **I** made my choices	**Strategy A somewhat better** describes how **I** made my choices	**Both strategies equally** describe how **I** made my choices	**Strategy B somewhat better** describes how **I** made my choices	**Strategy B much better** describes how **I** made my choices

Continue

Binary weights heuristic:

Next, we're going to describe another two big-picture strategies -- **Strategy C** and **Strategy D** -- that a person could have used to make choices in Section 1.

< Previous Next >

Strategies C and **D** are about how a person weighed together the features of the homes they cared about when making their choices.

< Previous Next >

A person using **Strategy C** cared about some features of the homes and didn't care about others. Critically, for the features they did care about, they **cared about them all equally**.

For instance, if the three features the person cared about were traffic, size of the home, and noise pollution, they cared about them *equally* when making their choices. Their thinking might look like this:

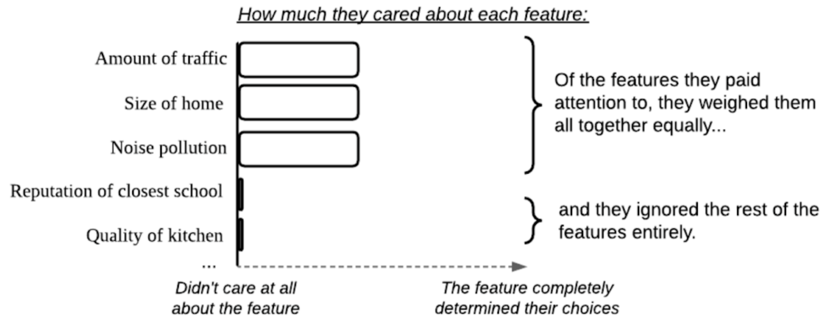

Of course, the person might have cared about different features (or a different number of features) than the ones in this example. Which features they cared about doesn't matter. The point is that, for the features they *did* care about, they cared about them all an equal amount.

< Previous Next >

Another way to put it is that, for **Strategy C**, the person either cared about a feature or didn't. There was no in-between (e.g. they didn't care about some features 'a little' versus 'a lot').

< Previous Next >

In contrast, a person using **Strategy D** placed a **different amount of importance** on **different features they cared about**.

For instance, if the three features the person considered were traffic, size of the home, and noise pollution, they cared about some of those features more than others when making their choices. Their thinking might look like this:

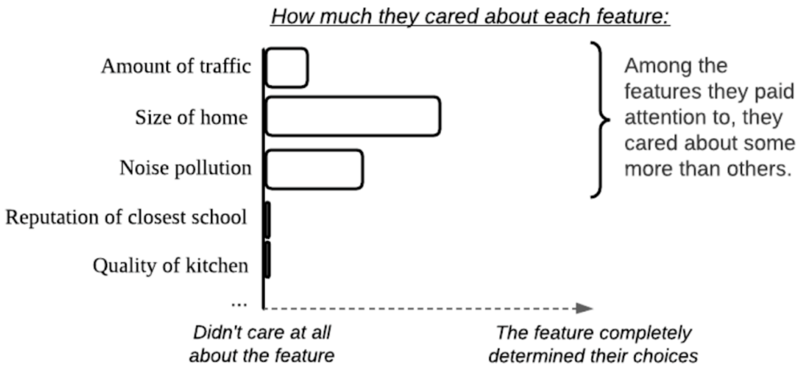

Of course, they might have cared about different features (or a different number of features) than the ones in this example. Which features they cared about doesn't matter. The point is that, for the features they *did* care about, they cared about some more than others.

< Previous Next >

Another way to put it is that, for **Strategy D**, the person cared about some features a little and some features a lot.

< Previous   Next >

Here are the two strategies described again, for reference.

**Strategy C:** For the features the person cared about, the person **cared about them all equally**. (In other words, the person either cared about a feature or they didn't; there was no in-between.)	**Strategy D:** For the features the person cared about, the person **placed a different amount of importance on different ones**. (In other words, the person might have cared about some features a little and some a lot.)

When you feel like you've understood the two strategies, press 'Continue'.

Continue

Now, read the following description of a person, and answer: **Which of the two strategies better reflects how this person made their choices?**

Person description: When making her choices between homes in Section 1, Leila cared about two features: the amount of noise pollution and the amount of traffic. She cared about both of those two features an equal amount. To decide which home was better overall, she compared how much better each home was on those two features.

**Strategy C much better** describes how this person made their choices	**Strategy C somewhat better** describes how this person made their choices	**Both strategies equally** describe how this person made their choices	**Strategy D somewhat better** describes how this person made their choices	**Strategy D much better** describes how this person made their choices

Continue

Now, read this description of a different person, and answer again: **Which of the two strategies better reflects how this person made their choices?**

Person description: When making her choices between homes in Section 2, Monica cared about four features: the size of the home, the size of the yard, the amount of traffic, and the amount of noise pollution. She cared about the size of the home and yard the most, but she also cared a little about the other two features.

**Strategy C much better** describes how this person made their choices	**Strategy C somewhat better** describes how this person made their choices	**Both strategies equally** describe how this person made their choices	**Strategy D somewhat better** describes how this person made their choices	**Strategy D much better** describes how this person made their choices

Continue

Next, we want to know: Which of the two strategies better reflects how **YOU** made **YOUR** choices in Section 1 of the task?

**Strategy C much better** describes how **I** made my choices	**Strategy C somewhat better** describes how **I** made my choices	**Both strategies equally** describe how **I** made my choices	**Strategy D somewhat better** describes how **I** made my choices	**Strategy D much better** describes how **I** made my choices

Continue

Binary attribute value heuristic:

Finally, we're going to describe a last two big-picture strategies -- **Strategy E** and **Strategy F** -- that a person could have used to make choices in Section 1.

< Previous Next >

Strategies E and **F** are about how a person compared the two homes on each feature.

< Previous Next >

A person using **Strategy E** compared the homes on each feature based just on **which** home was better or worse on that feature, but NOT **how much** better or worse it was.

For instance, if the person was considering the size of each home, they only checked *which* home was bigger; they didn't care about how *MUCH* bigger it was. Similarly, if the person was considering the amount of traffic near each home, they only checked which home had more traffic; they didn't care about how *much more* traffic it had. Their thinking might look like this:

	Home A	Home B
Size	1200 sq ft	1000 sq ft
...	...	...
...	...	...

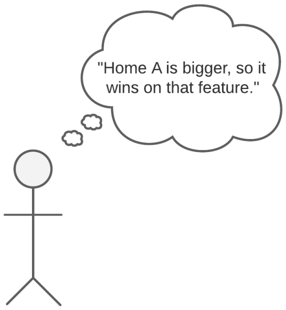

In this case, it wouldn't matter whether Home A was 200 square feet bigger or 1000 square feet bigger; the person would treat them the same. All that matters to this person is that Home A is bigger.

Of course, the person might have cared about different features than the ones in this example. But the point is that, for the features they did care about, they only considered which home was better or worse on each of those features (not how *much* better or worse it was).

< Previous Next >

Another way to put it is that, for **Strategy E**, the person just checked whether a home 'won' on each feature, and didn't care about whether it won by a lot or a little.

< Previous Next >

In contrast, a person using **Strategy F** went beyond checking which home was better or worse on each feature, and instead cared about **how much** better or worse it was.

For instance, if the person was considering the size of each home or amount of traffic near each home, they cared about how *much* bigger the bigger home was, or how *much more* traffic it had. Their thinking might look like this:

	**Home A**	**Home B**
Size	1200 sq ft	1000 sq ft
...	...	...
...	...	...

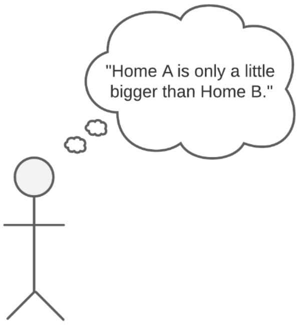

	**Home A**	**Home B**
Size	1800 sq ft	600 sq ft
...	...	...
...	...	...

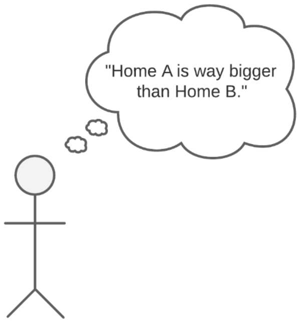

Of course, the person might have cared about different features than the ones in this example. But the point is that, for the features they did care about, they considered how *much* better or worse each home was on those features.

< Previous   Next >

Here are the two strategies described again, for reference.

**Strategy E:** When the person compared the two homes on a feature, they only checked **which** home was better or worse on that feature (i.e. which home 'won' that feature), and didn't care about how much better or worse it was on that feature.	**Strategy F:** When the person compared two homes on a feature, they cared about **how much** better or worse each home was on that feature.

When you feel like you've understood the two strategies, press 'Continue'.

Continue

Now, read the following description of a person, and answer: **Which of the two strategies better reflects how this person made their choices?**

Person description: When making his choices between homes in Section 1, Xavier cared about three features: the size of the home, the amount of traffic, and the closeness to parks. He checked which home was better on each feature (i.e. which home 'won' on each feature), and chose the home that 'won' on a majority of the three features.

**Strategy E much better** describes how this person made their choices	**Strategy E somewhat better** describes how this person made their choices	**Both strategies equally** describe how this person made their choices	**Strategy F somewhat better** describes how this person made their choices	**Strategy F much better** describes how this person made their choices

Continue

Now, read this description of a different person, and answer again: **Which of the two strategies better reflects how this person made their choices?**

Person description: When making his choices between homes in Section 1, Joe cared about three features: the year the building was built, the closeness to parks, and the size of the yard. He considered the difference between the two homes on each feature (e.g. a 100 sq. ft. yard versus a 400 sq. ft. yard = a 300 sq. ft. difference), and then weighed those differences together to figure out which home was better overall.

**Strategy E much better** describes how this person made their choices	**Strategy E somewhat better** describes how this person made their choices	**Both strategies equally** describe how this person made their choices	**Strategy F somewhat better** describes how this person made their choices	**Strategy F much better** describes how this person made their choices

Continue

Next, we want to know: Which of the two strategies better reflects how **YOU** made **YOUR** choices in Section 1 of the task?

**Strategy E much better** describes how **I** made my choices	**Strategy E somewhat better** describes how **I** made my choices	**Both strategies equally** describe how **I** made my choices	**Strategy F somewhat better** describes how **I** made my choices	**Strategy F much better** describes how **I** made my choices

Continue

In Study 1A, to measure the direction of participants' self-reported weights (e.g., whether they preferred larger or smaller homes), we included in Part 2 of the ACP task the following questions for each attribute:

Noise pollution

This feature could have taken on different values, such as: Very Low, Low, Moderate, High, Very High

When making your choices, which of these values would you have **most** preferred for 'Noise pollution'?

○ Very Low ○ Low ○ Moderate ○ High ○ Very High

Continue

Noise pollution

This feature could have taken on different values, such as: Very Low, Low, Moderate, High, Very High

When making your choices, which of these values would you have **least** preferred for 'Noise pollution'?

○ Very Low ○ Low ○ Moderate ○ High ○ Very High

Continue

For attributes with continuous values (e.g., home size), we had participants rank a subset of the possible values sampled uniformly across the space. We counted the participants' self-reported attribute direction as positive if their most preferred value was greater than their least preferred value, and negative if it was the reverse. (If a participant reported the same attribute value as both their most and least preferred for any attribute which they had reported a weight greater than 0.1 on, they were excluded from analysis.)

We found that participants were often confused by this question. Hence, in all subsequent studies, we asked the following set of questions instead (for each attribute):

Now imagine another two movies, which were again identical in every way except that they had different scores for the feature 'Humor'.

For one of the movies, 'Humor' had a score of **Very Bad**. For the other movie, 'Humor' had a score of **Bad**.

Which of these two movies would you have chosen?

○ The movie where 'Humor' was **Very Bad** ○ The movie where 'Humor' was **Bad**

Continue

Now imagine another two movies, which were again identical in every way except that they had different scores for the feature 'Humor'.

For one of the movies, 'Humor' had a score of **Bad**. For the other movie, 'Humor' had a score of **Moderate**.

Which of these two movies would you have chosen?

○ The movie where 'Humor' was **Bad** ○ The movie where 'Humor' was **Moderate**

Continue

Now imagine another two movies, which were again identical in every way except that they had different scores for the feature 'Humor'.

For one of the movies, 'Humor' had a score of **Moderate**. For the other movie, 'Humor' had a score of **Good**.

Which of these two movies would you have chosen?

○ The movie where 'Humor' was **Moderate** ○ The movie where 'Humor' was **Good**

Continue

Now imagine another two movies, which were again identical in every way except that they had different scores for the feature 'Humor'.

For one of the movies, 'Humor' had a score of **Moderate**. For the other movie, 'Humor' had a score of **Good**.

Which of these two movies would you have chosen?

○ The movie where 'Humor' was **Moderate** ○ The movie where 'Humor' was **Good**

Continue

Now imagine another two movies, which were again identical in every way except that they had different scores for the feature 'Humor'.

For one of the movies, 'Humor' had a score of **Good**. For the other movie, 'Humor' had a score of **Very Good**.

Which of these two movies would you have chosen?

○ The movie where 'Humor' was **Good** ○ The movie where 'Humor' was **Very Good**

Continue

Imagine that, in Section 1, you'd been given a choice between two movies which were identical in every way, EXCEPT that they had different scores for the feature 'Humor'.

For one of the movies, 'Humor' had a score of **Very Bad**. For the other movie, 'Humor' had a score of **Very Good**.

Which of these two movies would you have chosen?

○ The movie where 'Humor' was **Very Bad** ○ The movie where 'Humor' was **Very Good**

Continue

For the text of all other instructions and questions, see the experiment code posted online.

1.3. Additional questions and individual-difference battery

In Part 2, after the heuristic and attribute weight self-report sections, we gave participants four additional questions. First, we gave them another free-response question, asking them whether, after answering all the prior questions, their self-description of their strategy had changed at all. (We do not analyze the free-response questions in this manuscript; we just report them here for completeness.) Second, we asked them the degree to which they believed their use of heuristics remained consistent throughout the choice trials in Part 1. Third, we asked them the degree to

which they believed their attribute weights remained consistent throughout Part 1. Fourth, we asked them to report the degree to which the questions in Part 2 allowed them to accurately describe their choice process. The latter three questions are used in quality control checks, described below.

After the ACP task (at the end of the first session and throughout the second session), we gave participants the individual difference measures in Table S1.

Target Construct	Measure	Studies included
Confidence in self-reports	How confident are you that, when answering the questions in Section 2 of the task, you knew exactly how you made your choices in Section 1? (continuous slider)	All
Satisfaction with choices	How satisfied are you with the choices you made between homes in Section 1 of the task? Do you think you made the best choices for yourself? (continuous slider)	All
Strategy for making self-reports	In Section 2 of this study, we asked you questions about how you made your choices in Section 1. How did you answer those questions in Section 2? In other words, how do you know what your Section 1 choice process was? (free response box)	All
Attention paid during study (self-reported)	How attentive were you throughout the experiment? (Please be honest; we understand that it's hard to focus throughout the study, and we will accept your submission no matter how you answer.) (continuous	All

	slider)	
Experience with choice domain	In your life, how much time have you spent thinking about which <houses or apartments to rent> <movies to watch>? (continuous slider)	All
Experience with multi-attribute choice studies	How many studies like this one (where you had to choose between two options which varied on many features) have you participated in before? (button options: 0, 1-5, 5-10, 10-15, 15+)	All
Meditation experience	Have you tried mindfulness meditation in the past? (yes/no)<br><br>If yes: For how many years have you practiced mindfulness meditation? (options: 'Less than 1 year', 'Between 1-5 years', 'More than 5 years')<br><br>How many times have you practiced mindfulness meditation in the past 30 days? (text box)<br><br>In the past 30 days, on average, how many minutes did you spend meditating in each session? (text box)<br><br>Have you ever received mindfulness training or participated in a mindfulness/acceptance-based therapy program? (yes/no)	All

	Select the option that best describes your experience with mindfulness meditation. (options: 'I am relatively new to mindfulness meditation.', 'I have a moderate amount of experience with mindfulness meditation.', 'I have an extensive amount of experience with mindfulness meditation.')	
Gender	Do you describe yourself as a man, a woman, or in some other way? (options: 'Man', 'Woman','Some other way', 'Prefer not to say')	All
Age	Text box	All
Race	Race/ethnicity: Which categories below describe you? Please select all that apply. (check boxes, with options: 'White', 'Hispanic, Latinx, or Spanish origin', 'Black or African American', 'Asian', 'American Indian or Alaska Native', 'Middle Eastern or North African', 'Native Hawaiian or Other Pacific Islander', 'Some other race, ethnicity, or origin', 'Prefer not to say')	All
Native language	Is English your first language? (options: 'Yes', 'No', 'Prefer not to say')	All
Education level	What is the highest level of education that you have completed? (options: "Some high school","High school", "Some college", "2	All

	year degree", "4 year degree", "Postgraduate/Professional degree/other", "Prefer not to say")	
Income level	What was the total amount of income you earned during the last year? (options: 'Under $5,000', '$5,000 - $10,000', '$10,000 - $15,000', '$15,000 - $25,000', '$25,000 - $35,000', '$35,000 - $50,000', '$50,000 - $65,000', '$65,000 - $80,000', '$80,000 - $100,000', 'Over $100,000', 'Prefer not to say')	All
Attentional capacity / efficiency	Attention network test[4]	1A, 1B, 2, 3A
Cognitive / reasoning ability	International cognitive ability resource[5]	1A, 1B, 2, 3A
Decision style (intuitive vs deliberative)	Decision styles scale[6]	1A, 1B, 2, 3A
Self-reported attentional control	Attentional control scale[7]	1A, 1B, 2, 3A
Trait mindfulness	Cognitive Affective Mindfulness Scale – Revised[8]	1A, 1B, 2, 3A
Self-reported introspective capacity and interest	Self-reflective insight scale[9]	1A, 1B, 2, 3A
Self-reported interoceptive awareness	Multidimensional scale of interoceptive awareness[10]	1A, 1B, 2, 3A

Table S1: List of measures collected after the ACP task.

1.4. Self-reported method of answering self-report questions

As an additional question in the final section of the ACP task, we asked participants to report how they believe they answered the self-reports questions in Part 2 – i.e., how they knew their own choice process. The exact question text was: "In Section 2 of this study, we asked you questions about how you made your choices in Section 1. How did you answer those questions in Section 2? In other words, how do you know what your Section 1 choice process was?" Though we do not analyze these open-ended text data in this paper, we note here that, anecdotally, not a single participant reported doing any kind of sophisticated inference about their own attitudes (e.g., by observing their own choices, recounting past memories of similar choices, recruiting complex lay theories). The most common responses were akin to "I looked into my mind". Of course, participants could be wrong about how they are producing their self-reports. Nonetheless, these data indicate that participants are not consciously or intentionally employing inferential mechanisms to infer their choice processes.

1.5. How does the ACP task differ from prior work on awareness in multi-attribute choice?

The idea to leverage multi-attribute choice to examine choice process awareness is not new: A set of studies from the 1970s-1980s examined people's awareness of their attribute weights in multi-attribute judgments using a paradigm that forms the foundation of the present ACP task. In one study, for instance, participants judged the appeal of colleges that varied on tuition, size, etc[11]. Participants then reported how much they believed each attribute had influenced their choices, and their awareness was scored by comparing these self-reports to coefficients obtained from a linear regression analysis – i.e., regressing their judgments on all the attributes simultaneously. Studies using paradigms like this produced inconsistent results, with some finding significant awareness[12–18] and others not[19–24].

These past studies, however, had significant methodological limitations which may have contributed to their mixed results. First and foremost, they employed only one model of people's choice process: They assumed that people always used the classically-rational model of multi-attribute choice[25]. However, as described above, people often employ heuristic simplifications that substantially alter their choice process[26]. By assuming everyone used the rational process, past paradigms likely computed awareness scores that were biased and incomplete, and missed the opportunity to obtain richer and more holistic measures of awareness. Moreover, these studies typically only asked participants to make numerical judgments of choice options one at a time (e.g. "How likely would you be to attend this college?"), rather than actual choices between options (e.g., "Which of these colleges would you attend?"), potentially missing important aspects of the dynamics in actual choice contexts. Finally, past paradigms used outdated model-fitting techniques; used only hypothetical (i.e., not incentive-compatible) choices; and tended to use small convenience samples of U.S. undergraduates. The lack of clarity resulting from these past paradigms highlights the need for an updated measure – such as our ACP task – that can rigorously quantify individual-level awareness of key aspects of people's multi-attribute choice processes.

1.6. Details of Study 4 (expert prediction study)

We recruited decision scientists for Study 4 in two ways: in person at the Society for Judgment and Decision Making (SJDM) 2024 conference, and via the SJDM email listserv. The in-person recruiting was conducted by passing out flyers and asking conference attendees directly. We recruited from the listserv with the following email:

Dear SJDM community,

We are eliciting forecasts from behavioral scientists about what they believe the results of an experiment on introspection would be. If you are willing to take a short survey, please follow the link below. ***The survey takes only ~5 minutes, and your participation is extremely appreciated!*** *We would love for anyone with (or working on) a PhD related to behavioral science to participate.*

Survey link: <LINK HERE>

Thanks so much.

Participants were not compensated. The survey itself described the home-rental variant of the ACP task in the following way (full materials can be found on our OSF page):

The task is called the "Awareness of Choice Processes" (ACP) task. It has two parts.

In **Part 1**, participants are repeatedly shown two different homes and asked to **choose which one they'd rather rent**. Here's an example choice:

	Home A	Home B
**Noise pollution**	High	Low
**Quality of kitchen**	Very Good	Bad
**Quality of heat/AC system**	Moderate	Very Good
**Year building was built**	1993	1953
**Amount of traffic**	A Little	A Lot
**Closeness to parks**	20 min away	30 min away
**Size of home**	1700 sq ft	800 sq ft
**Size of yard**	100 sq ft	250 sq ft
**Reputation of closest school**	Very Good	Moderate

On each trial, participants see two new homes and choose which one they prefer. Before starting, participants are told to pay attention to how they're making choices.

Then, in **Part 2**, participants are asked to **report how much weight they think they placed on each attribute of the homes** when making their choices in Part 1**.** Here's what the question looked like:

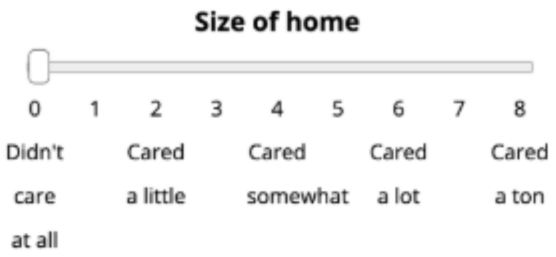

How much did you care about this feature when making your decisions?

Finally, after participants completed the study, we fit a computational model to their choices and **identified the *actual* weight they had placed on each attribute.**

Then, we quantified each participant's accuracy by comparing their *self-reported* attribute weights to these *best-fit* attribute weights. Specifically, we computed the Pearson correlation between the self-reported weights and the best-fit weights across all attributes. We call this value the "subject-level correlation". Intuitively, a person who knew their own attribute weights really well would have a high correlation, and a person who did not know their own attribute weights would have a low correlation.

Imagine we ran this task with 300 U.S. subjects on Prolific, with a sample that was nationally representative for age, race, and gender. What do you predict would be the **average subject-level correlation** between the self-reported and best-fit attribute weights?

0 0.1 0.2 0.3 0.4 0.5 0.6 0.7 0.8 0.9 1

I predict the average subject-level correlation would be:

Prior research has shown that people often use heuristics to simplify their choice processes. In addition to asking participants about their attribute weights, we also asked to report **whether they think they employed heuristics while making their choices.**

Specifically, we asked them about **three potential heuristics** they could have used. They were:

Heuristic #1 ("take the best" heuristic): Instead of integrating together multiple attributes, people sometimes choose based on just a single attribute.

Heuristic #2 ("in or out" heuristic): Instead of assigning graded weights to each attribute, people sometimes just represent some attributes as "in" and some as "out" of consideration, and weigh all the "in" ones equally.

Heuristic #3 ("attribute comparison" heuristic): Instead of representing the true value of each option on each attribute (e.g., "Home A is 2000 sq. ft."), people sometimes just represent which option is better on each attribute (e.g., "Home A is bigger").

We described each heuristic in intuitive language, and asked participants to **report whether they think they used that heuristic or not.**

Then, after participants completed the study, we again fit a computational model to their choices and **identified whether participants in fact used each heuristic.**

What percentage of participants do you think correctly reported whether they used the heuristic or not, for all three heuristics? (If participants were choosing randomly, they would get all three heuristics right 17% of the time.)

0 10 20 30 40 50 60 70 80 90 100

I predict that the % of participants reporting all three heuristics correctly would be:

We also collected information about, demographics, role, fields of study, and whether they had heard of the ACP task before.

2. Analysis

2.1. Modeling procedure

As described in the Methods section, the three heuristics in Table 1 combine to form six potential overall models of people's choice process[27]: (1) none of the three heuristics (the "classically rational" model); (2) just the binary attributes heuristic ("bin-atts"); (3) just the binary weights heuristic ("bin-wts"); (4) both the binary weights and binary attributes heuristic ("bin-atts-wts"); (5) just the single attribute heuristic ("single-att"); and (6) the single attribute and binary attributes heuristic ("single-bin-att"). Call this set of models $AllModels$ = {rational, bin-atts, bin-wts, bin-atts-wts, single-att, single-bin-att}.

In all models, agents compute overall option values for each of the two choice options (labeled $OptVal(j)$ for option $j \in \{1,2\}$), and then convert them into choices via a softmax function with inverse temperature parameter β (Equation 1):

$$Prob(choice = j) = \frac{e^{\beta * OptVal(j)}}{e^{\beta * OptVal(j)} + e^{\beta * OptVal(j')}}$$

where j' is shorthand for the other choice option (of $\{1,2\}$) not equal to j.

The models differed in terms of how those overall values were computed. The rational model assigned graded weights $w_{1...9} \in [-1,1]$ for each of the 9 attributes, and represented the values of each attribute on each option (labeled $AttVal(i,j)$ for attribute *i* of option *j*), scaled such that the range of each attribute was 0-1. Then, on each choice trial, it linearly combines the weights and attribute values to compute overall option values (Equation 2):

$$OptVal(option\, j) = \sum_{i=1}^{9} w_i * AttVal(i,j)$$

The bin-atts model replaces $AttVal\ (i,j)$ in Equation 2 with $bigger(AttVal(i,j))$, which equals 1 if $AttVal(i,j) > AttVal(i,j')$ and 0 otherwise. The bin-wts model replaces $w_{1...9} \in [-1,1]$ with $w_{1...9} \in \{-1,0,1\}$. The bin-atts-wts model does both. The single-att model uses $w_{1...9} \in \{-1,0,1\}$ but with the constraint that exactly one $w \in w_{1...9}$ had to be nonzero, and the rest zero. (The single-att model can be equivalently "compactly" parameterized by a parameter $u \in \{1, ..., 9\}$ which indicates which attribute was the attribute used, and then a parameter $sign \in \{-1,1\}$ which indicates the sign of the weight. The long parameterization is useful for comparing the model to others; the compact parameterization is useful for fitting the model. Our modeling procedure switches back and forth between these parameterizations as needed.) Finally, the single-bin-att model uses those weight constraints along with $AttVal\ (i,j)$ in Equation S2 replaced with $bigger(AttVal(i,j))$. (Note that the single-att and single-bin-att models exhibit different behavior because of the probabilistic nature of the softmax function; even when choosing based on only one attribute, it matters whether the agent is sensitive or not to the magnitude of the difference in attribute values between the two options.)

Thus, the parameters for each model are β and $w_{1\ldots9}$ (or $\beta, u,$ and $sign$ in the compact parameterization of the single-att models). We assume a uniform prior on all weight parameters (a continuous uniform prior between -1 and 1 for the graded weights, and a discrete uniform prior for the binary and single-att weights), and a Gamma(4, 1) prior for the inverse temperature parameter.

We fit the rational and bin-atts models in Stan[28], with 4 chains and 2000 iterations per chain (using the R package *RStan*). From each model, we extracted the maximum a posteriori weights and the marginal likelihood (i.e., for model $k \in AllModels$, $Prob(choices \mid model\ =\ k)$) using bridge sampling[29]).

Unfortunately, Stan cannot fit models with discrete free parameters. Hence, to fit the bin-wts, bin-atts-wts, single-att, and single-bin-att models, we had to implement a manual workaround. We first describe how we computed the single-att models, and then turn to the more complex bin-wts models.

The discrete parameters of each single-att model have 18 possible value sets they could take on: a value of 1 or -1 for each of the 9 attribute weights (e.g., $w_{1\ldots9} = \{1,0,\ldots,0\}$, or $\{-1,0,\ldots,0\}$, or $\{0,1,0,\ldots,0\}$, and so on). Assuming one of these sets (e.g., assuming $w_{1\ldots9} = \{1,0,\ldots,0\}$) yields a "sub-model" with just one free parameter: the inverse temperature β. For each of the 18 sub-models, we manually compute (a) the optimal value for β with the R function *optimize*, and (b) the marginal likelihood for that sub-model by integrating out β (from 0 to 20, since β values greater than 20 contributed an insignificant amount to the posterior). Then, zooming back out, we can extract the best-fitting parameters of the entire single-att model by seeing which sub-model produced the highest posterior probability after optimizing for β (e.g., if the sub-model assuming $w_{1\ldots9} = \{1,0,\ldots,0\}$ produced the highest posterior value with an optimal $\beta = 1.75$, then the best-fit parameters for the single-att model are $w_{1\ldots9} = \{1,0,\ldots,0\}$ and $\beta = 1.75$).

Finally, we can extract the marginal likelihood of the entire single-att model by averaging the marginal likelihoods of all the sub-models. (Note that this analysis assumes a uniform prior across the discrete parameters, and hence a uniform prior across sub-models.) To see why this is the case, recall that the marginal likelihood is obtained by taking the model's likelihood function and integrating out the model's free parameters Θ:

$$Prob(choices \mid model = m) = \int_{\theta} Prob(choices \mid model = m, \Theta = \theta) * Prob(\Theta = \theta) d\theta$$

When the parameters can be decomposed into the continuous parameters Θ_C and discrete parameters Θ_D, this equation can be rewritten as:

$$Prob(choices \mid model = m)$$

$$= \sum_{\theta_D} (Prob(\Theta_D = \theta_D) * \int_{\theta_C} Prob(choices \mid model = m, \Theta_C = \theta_C, \Theta_D = \theta_D) * Prob(\Theta_C = \theta_C) d\theta_C)$$

But the integral term is just the marginal likelihood of each sub-model, and we assumed a uniform prior over discrete parameters (i.e., $Prob(\Theta_D = \theta_D) = \frac{1}{|\Theta_D|}$ is constant). Hence, we can obtain the marginal likelihood of the entire model by averaging the marginal likelihoods of the sub-models.

The procedure for the bin-wts and bin-atts-wts models is similar, with one modification. In the case of these models, the number of possible value sets for the discrete parameters is much larger: $3^9 = 19{,}683$, to be exact (because each of 9 weights can take on any of three values $\{-1,0,1\}$). Computing the best-fit β and marginal likelihood for each of these sub-models would be very computationally expensive. Fortunately, most of these sub-models produce model fits that are uniformly very bad (because any random combination of weights is likely to fit a person's choices very poorly); it is only the best sub-models that contribute meaningfully to the results. Hence, we can drastically simplify this model-fitting procedure. We first estimate which sub-models are going to be the best by rapidly computing the posterior probability of all discrete value sets assuming $\beta = 1$. For the top 1000 of these value sets with the highest posterior probabilities, we manually compute the best-fit β and marginal likelihood using the same procedure described above. (The posterior probabilities and marginal likelihoods of the sub-models asymptoted to a uniformly low value well within the top 1000.) Then, to estimate the average marginal likelihood of the other 18,683 sub-models, we randomly sample 100 of them, manually compute their marginal likelihoods, and then sample with replacement from these 100 to estimate the other marginal likelihoods of the other 18,583. Finally, zooming out, the best-fit parameters of the entire model are the β and weights of the sub-model (of the initial 1000 tested) with the highest posterior probability; and we obtain the marginal likelihood of the entire model by averaging over the marginal likelihoods of all sub-models (the top 1000 that were computed precisely, and then the bottom 18,683 which were estimated via the sampling procedure described above). Extensive testing demonstrated that this procedure produces highly accurate results, including in the simulations described in detail below.

After obtaining the marginal likelihoods for each model, we can compute the posterior probability of each model m (assuming a uniform prior over models) by normalizing the marginal likelihoods (Equation 3):

$$Prob(model = m \mid choices) = \frac{Prob(choices \mid model = m)}{\sum_{k \in AllModels} Prob(choices \mid model = k)}$$

As described in Methods, we assessed the appropriateness of the model fits in several ways. First, we assessed the following Stan model diagnostics to identify issues with the model fits: effective sample size (ESS), R-hat, tree depth saturations, energy Bayesian fraction of missing information (E-BFMI), and number of divergences[30]. Then, we used leave-one-out cross-validation (approximated via the "loo" package[31]) to assess overall model fits; this method gives us the "expected log predictive density", or (roughly) the expected probability density assigned to out-of-sample choices by the model.

Finally, we computed two types of split-half reliability estimates: one splitting the data into even/odd trials and one splitting into first-half/second-half of trials. The even/odd trial split allowed us to test the basic split-half reliability of the model-fitting estimates; the first-half/second-half split allowed us to test whether that reliability was lessened because participants changed their decision process over the course of the task. To implement this procedure, we split the data accordingly, refit the models to each half using the same procedure as above, and then correlated the various resulting model estimates across the two halves.

2.2. Computing the probability of each heuristic

The model fits were done over full models, which were combinations of the various heuristics in Table 1 of the main text. From these model fits, we extracted the probability that each participant used each individual heuristic, independent of the others, using Bayesian model family comparison[32]. For each heuristic $h \in$ {single attribute, binary weights, binary attribute values}, let $family(h)$ be the subset of $AllModels$ that used heuristic h, and $\neg family(h)$ be the subset of models that did not. So, for $h =$ single attribute heuristic, $family(h)$ would be {single-att, single-bin-att}, and $\neg family(h)$ would be {rational, bin-atts, bin-wts, bin-atts-wts}. For the binary attribute values heuristic, $family(h)$ would be {bin-atts, bin-atts-wts, single-bin-att}, and $\neg family(h)$ would be {rational, bin-wts, single-att}. The binary weights heuristic is only defined and recoverable in the context of multiple attributes, because models using the single attribute heuristic are equivalent with binary or graded weights; hence, for it we consider $family(h)$ to be {bin-wts, bin-atts-wts}, and $\neg family(h)$ to be {rational, bin-atts}, ignoring the single-attribute models.

The key to Bayesian family comparison is to set a uniform prior over *families* of models, so as not to bias inference in favor of heuristics which happened to be implemented in more individual models[32]. When analyzing heuristic h, this is accomplished by setting the prior on each individual model within $family(h)$ to be (Equation 4):

$$Prob(model = m) = \begin{cases} \dfrac{1}{2|family(h)|} \; if\ m \in family(h) \\ \dfrac{1}{2|\neg family(h)|} \; if\ m \in \neg family(h) \end{cases}$$

where $|family(h)|$ is the number of models in $family(h)$. This prior ensures that the total priors within the $family(h)$ and $\neg family(h)$ subsets are each 0.5. With this prior, the posterior probability of model $m \in family(h)$ is:

$$Prob(model = m \mid choices) = \frac{Prob(choices \mid model = m) * Prob(model = m)}{\sum_{i \in AllModels} Prob(choices \mid model = i) * Prob(model = i)}$$

By decomposing the denominator into $family(h)$ and $\neg family(h)$ and filling in the priors from Equation 4, we get:

$$Prob(model = m \mid choices) = \frac{\frac{1}{2|family(h)|} Prob(choices \mid model = m)}{\sum_{i \in family(h)} \frac{1}{2|family(h)|} Prob(choices \mid model = i) + \sum_{i \in \neg family(h)} \frac{1}{2|\neg family(h)|} Prob(choices \mid model = i)}$$

Multiplying both the numerator and denominator by 2 and rearranging terms, we get:

$$Prob(model = m \mid choices) = \frac{\frac{1}{|family(h)|} Prob(choices \mid model = m)}{\frac{1}{|family(h)|} \sum_{i \in family(h)} Prob(choices \mid model = i) + \frac{1}{|\neg family(h)|} \sum_{i \in \neg family(h)} Prob(choices \mid model = i)}$$

Finally, to get the probability that the participant's model came from $family(h)$, we simply sum the posteriors of each model in the family (Equation 5):

$$Prob(model \in family(h)) = \sum_{i \in family(h)} Prob(model = i \mid choices) = \frac{\frac{1}{|family(h)|} \sum_{i \in family(h)} Prob(choices \mid model = i)}{\frac{1}{|family(h)|} \sum_{i \in family(h)} Prob(choices \mid model = i) + \frac{1}{|\neg family(h)|} \sum_{i \in \neg family(h)} Prob(choices \mid model = i)}$$

In other words, the probability that the participant's model came from $family(h)$ – and thus the participant used heuristic h – is the average of the marginal likelihoods of all the models in $family(h)$ divided by the sum of that average and the average of the marginal likelihoods in $\neg family(h)$.

2.3. Simulation procedure

To test whether our model-fitting procedure worked in principle, we simulated data in the ACP task from 6000 agents – 1000 agents generated using each of the six choice

models described above – with weights and inverse temperatures randomly sampled via the same priors used in the model-fitting. Then, we ran our model-fitting procedure on these simulated data, and compared the results to the ground truth for each agent.

The model-fitting was highly accurate at recovering agents' weights: The average agent-level correlation between the best-fit weights and the true weights was, for the six choice models described above (in order): 0.97, 0.96, 1.0, 1.0, 1.0, 1.0 (overall average: 0.99). The model-fitting was also accurate at recovering agents' overall choice models and individual heuristics used. It correctly identified whether the agent was using each of the three heuristics from Table 1 99.8%, 85.8%, and 91.4% of the time (respectively), and it identified the overall correct model 83% of the time.

2.4. Regression analyses

For all analyses involving multiple data points per participant, we estimated mixed effect regressions using the R package *lme4*. To specify an optimal random effects structure, we adapted a procedure similar to that advocated by refs.[33,34]. We began by specifying a maximal structure, with random intercepts and all available random slopes for each participant. If there were convergence issues (a boundary/singular fit or a negative eigenvalue error), we first tried disallowing correlation between the random effects. If convergence issues persisted, we then removed any random effects that accounted for zero variance (after rounding to five decimal places). This procedure yields slightly different final structures for each reported regression; all final structures are detailed in the online analysis code. All *p* values were computed using the default Satterthwaite method in the package *lmerTest*[35,36].

2.5. Determining chance levels of accuracy measures

For several of the accuracy measures – specifically, the participant-level weight correlation, heuristic error, and reported model Bayes factor – it is not clear how to analytically derive the levels expected by chance. Hence, we identified chance levels via bootstrapping simulation. For each study, we randomly sampled 1000 participants with replacement from the study data and then simulated the level of each measure that would have been obtained if these participants had chosen randomly. For the weight correlations, we shuffled each sampled participant's reported weights across the nine attributes. For heuristic error, we assumed that participants were reporting an answer to each heuristic question uniformly at random across the scale. For the reported model Bayes factor, we assumed that participants were choosing randomly between the six choice models.

Supplementary discussion

3. Supplementary results for Studies 1-2

3.1. Participants

The sample sizes and demographic breakdown of participants are presented in Table S2. The first study session (run in all studies) lasted approximately an hour; the second study session (run in Studies 1A, 1B, 2, and 3A) lasted approximately 25 minutes. Participants were paid $12/hr for their participation (plus a $1 bonus for completing both sessions).

**Study**	1A	1B	2	3A	3B
**N recruited**	300	300	300	300	300
**N excluded**	63	49	65	88	91
**N in final sample**	237	251	235	212	209
**Gender**	52.7% F, 45.6% M, 1.7% Other	52.6% F, 43.4% M, 2.8% Other, 1.2% Prefer not to say	50.0% F, 44.7% M, 4.7% Other, 0.85% Prefer not to say	49.5% F, 47.6% M, 2.4% Other, 0.47% Prefer not to say	57.9% F, 39.7% M, 2.4% Other
**Age**	Mean = 46 years old, SD = 16	Mean = 39, SD = 14	Mean = 43, SD = 15	Mean = 46, SD = 16	Mean = 46, SD = 16
**Race**	0.85% American Indian or Alaska Native (AIAN), 5.5% Asian, 8.9% Black, 4.2% Latinx, 75% White, 5.5% Mixed race	5.6% Asian, 10.0% Black, 6.4% Latinx, 0.8% Middle Eastern or North African (MENA), 67.7% White, 8.3% Mixed race 0.4% Other,	3.8% Asian, 11.1% Black, 3.4% Latinx, 74.0% White, 6.85% Mixed race, 0.85% Prefer not to say	4.7% Asian, 11.8% Black, 3.3% Latinx, 72.6% White, 6.66% Mixed race, 0.94% Prefer not to say	0.48% AIAN, 5.7% Asian, 11.5% Black, 2.9% Latinx, 0.96% MENA, 71.3% White, 6.7% Mixed race, 0.48% Other,

		0.8% Prefer not to say			
**Education level**	0.84% some high school, 13.9% high school, 21.5% some college, 10.1% 2-year degree, 38.8% 4-year degree, 14.8% postgraduate degree	0.4% some high school, 15.5% high school, 26.7% some college, 10.0% 2-year degree, 36.7% 4-year degree, 10.0% postgraduate degree, 0.8% Prefer not to say	0.4% some high school, 10.6% high school, 17.9% some college, 14.9% 2-year degree, 36.2% 4-year degree, 20.0% postgraduate degree	0.5% some high school, 9.9% high school, 19.8% some college, 11.8% 2-year degree, 37.3% 4-year degree, 20.3% postgraduate degree, 0.47% Prefer not to say	14% high school, 29% some college, 13% 2-year degree, 33% 4-year degree, 11% postgraduate degree

Table S2: Sample sizes and demographic breakdown of participants.

3.2. Quality control checks

We ran a variety of quality control checks on our data, testing assumptions underlying the ACP task.

One assumption is that our models of participants' choice processes reasonably captured how the majority of our participants were making choices in the task. We tested this assumption in several ways. First, participants themselves believed that this was the case: When asked the extent to which they believed that the questions in Part 2 allowed them to accurately describe their choice process, participants responded with an average of 89.9 [88.3 - 91.4] out of 100 (Study 1A; in Study 1B, 88.8 [87.1 - 90.4]; in Study 2, 85.1 [82.9 - 87.2]). Second, our model fits were acceptable. For one, the Stan diagnostics for the best-fitting models all indicated valid model fits (see Table S3). Moreover, the best model for each participant had on average a cross-validated out-of-sample accuracy of 73.7% [72.2% - 75.1%] (Study 1A; in Study 1B, 72.9% [71.4% - 74.3%]; in Study 2, 71.3% [70.3% - 72.4%]) – indicating that the best-fitting models could predict 70-74% of participants' out-of-sample choices (compared to chance of 50%). In addition, the model-fitting results tended to be confident about which model was best for each participant; the average posterior probability of the best-fitting model was 0.75 [0.73 - 0.77] (compared to a chance probability of 1/6, or 0.167; in Study 1B, 0.77 [0.75 - 0.79]; in Study 2, 0.74 [0.71 - 0.76]). Finally, the model-fitting exhibited reasonable split-half reliability. The attribute weights fitted to even trials were moderately correlated at with the weights fitted to odd trials (Study 1A, $r = 0.77$ [0.75 - 0.79]; Study 1B: $r = 0.79$ [0.77 - 0.80]; Study 2: $r = 0.49$ [0.45 - 0.52]; all p's < .001). Similarly, the probabilities of each heuristic were moderately correlated between

even/odd trials (Study 1A, r = 0.59 [0.54 - 0.63]; Study 1B, r = 0.62 [0.58 - 0.66]; Study 2, r = 0.59 [0.54 - 0.64]; all p's < .001). Of course, these split-half reliabilities are far from perfect, further emphasizing that our accuracy estimates are lower bounds on participants' true accuracy.

	**Study 1A**	**Study 1B**	**Study 2**
**% of iterations ending with a divergence**	95% of subjects had no divergent iterations; average % divergent was 0.16%	94% of subjects had no divergent iterations; average % divergent was 0.18%	96% of subjects had no divergent iterations; average % divergent was 0.06%
**% of iterations saturating tree depth**	0%	0%	0%
**Energy Bayesian fraction of missing information**	100% of subjects had E-BFMI > 0.2	100% of subjects had E-BFMI > 0.2	100% of subjects had E-BFMI > 0.2
**Effective sample size**	100% of subjects had an effective sample size > 0.001 per iteration	100% of subjects had an effective sample size > 0.001 per iteration	100% of subjects had an effective sample size > 0.001 per iteration
**R-hat**	100% of subjects had R-hat < 1.1	100% of subjects had R-hat < 1.1	100% of subjects had R-hat < 1.1

Table S3: Diagnostic checks for the best-fitting model of each participant. For a description of each diagnostic, see ref.[30].

Another assumption is that people did not substantially change their model or attribute weights over the course of the choice trials. Participants reported this tended to be the case: When asked the extent to which their heuristics and attribute weights remained consistent throughout Part 1, they responded with an average of 81.0 [78.8 – 83.2] and 79.9 [77.7 – 82.2] out of 100, respectively (Study 1A; in Study 1B, 79 [76.6 - 81.4] and 76.2 [73.6 - 78.9]; in Study 2, 74.8 [72.4 - 77.3] and 69.9 [67.1 - 72.7]). Moreover, the split-half reliability of the model fits was similar when splitting on even/odd trials versus first half/second half of trials; across the three studies, the correlation between weights estimated from first half and second half was r = 0.77 [0.75 - 0.79], 0.74 [0.73 - 0.76], 0.44 [0.41 - 0.48], and the correlation between heuristic probabilities estimated from first half and second half was r = 0.53 [0.47 - 0.58], 0.52 [0.47 - 0.57], and 0.43 [0.36 - 0.49], indicating that participants' strategy did not change substantially from the first to the second half. (When excluding participants who reported <50% weight consistency, we still found average weight accuracies of 0.81 [0.77 - 0.85] (Study 1a), 0.88 [0.86 - 0.91]

(Study 1b), and 0.58 [0.53 - 0.63] (Study 2) – similar to the accuracies without exclusion. When excluding participants who reported <50% heuristic consistency, we still found average heuristic errors of 0.33 [0.31 - 0.36] (Study 1a), 0.32 [0.3 - 0.35] (Study 1b), and 0.27 [0.25 - 0.3] (Study 2) – again, similar to the error without exclusion.)

A third assumption is that participants understood the questions in Part 2. Participants got an average of 5.56 [5.47 – 5.64] out of 6 comprehension check questions correct (Study 1A; in Study 1B, 5.65 [5.57 - 5.72]; in Study 2, 5.51 [5.42 - 5.6]), and rarely reported confusion in a free-response feedback box we gave them at the end of the study.

A final assumption is that participants valued each attribute of the choice options in a way that monotonically increased or decreased across the attributes' scale. For instance, we assumed that participants in the home variant desired the potential values of "noise pollution" in a monotonic order – e.g., "Very Good" was better than "Good", which was better than "Good", which was better than "Moderate", which was better than "Bad", which was better than "Very Bad". This assumption was necessary to make the model-fitting tractable.

One way to test this assumption was to check if participants' self-reported preferences were monotonic, using the "weight direction" questions described at the end of Section 1.2 above. In Study 1A, we counted a participant's preference for that attribute as monotonic if they reported one end of the attribute scale as their most preferred and the other end as their least preferred. Averaging across participants, 75% [73% - 77%] of attributes were valued monotonically. In all other studies, we counted a participant's preference for each attribute as monotonic if their reported preferences were monotonic across the whole scale (e.g., they preferred Very Good > Good > Moderate > Bad > Very Bad). In Study 1B, 89% [88% - 91%] of attributes were valued monotonically; in Study 2, 93% [92% - 94%] were.

Thus, participants reported largely monotonic preferences. Of course, participants' self-reports about monotonicity could be wrong; non-monotonic preferences would simply show up as poorer model fits.

3.3. Plots for Study 1B

All the plots for Study 1B are presented in Fig. S1.

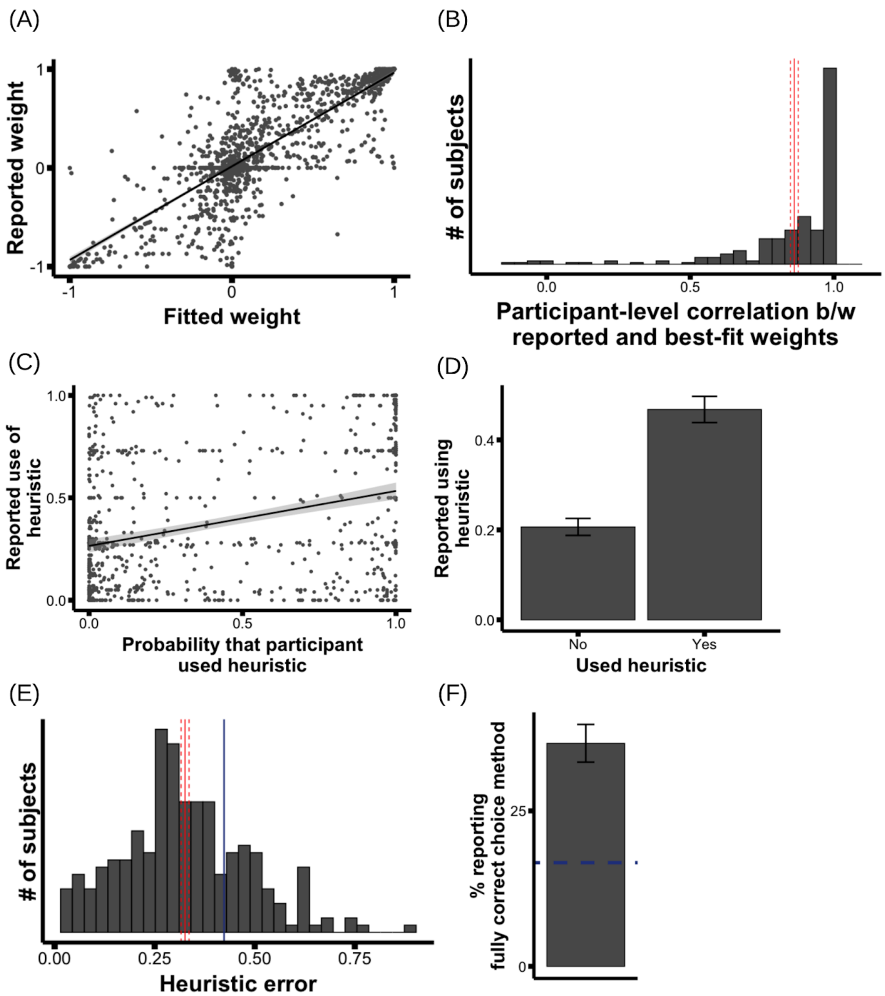

Figure S1: Plots for Study 1B. Weight accuracy measures (described in main text) are shown in (A)-(B); method accuracy measures are shown in (C)-(F). (A) and (C) show least-squares trend lines (with 95% confidence interval bands). Red solid lines indicate the sample mean, with the dashed lines showing the standard error of the mean (SEM); blue lines indicate chance. All error bars are SEM.

3.4. Descriptives of which heuristics & weights people used and reported using

To better contextualize all the results, it is helpful to know the raw extent to which participants reported using and actually used each heuristic. In Study 1A, when asked to report how well the three heuristics from Table 1 described their choice process (where 0 is no and 100 is yes), participants reported an average of 36.8 [32.7 - 40.9], 28.7 [24.7 - 32.7], and 45.0 [40.8 - 49.2] respectively. The modal participant reported using none of the heuristics, but there was substantial variation in reports. According to the model fits, the average probability that participants used each heuristic was 54.7 [48.8 - 60.6], 45.4 [40.6 - 50.3], 29.8 [25.7 - 33.9] respectively. The modal participant used the single-attribute heuristic (and not the other two), but again there was substantial variation.

Study 1B showed similar patterns. When asked to report how well the three heuristics described their choice process, participants reported an average of 34.6 [30.4 - 38.9], 30.7 [26.7 - 34.6], and 43.4 [39.4 - 47.4], respectively. The modal participant reported using none of the heuristics, with substantial variation. According to the model fits, the average probability that participants used each heuristic was 49.2 [43.4 - 54.9], 43.6 [38.9 - 48.2], and 28.1 [24 - 32.1], respectively. The modal participant used just the single-attribute heuristic, with substantial variation.

In Study 2, when asked to report how well the three heuristics described their choice process, participants reported an average of 23.5 [19.9 - 27.1], 32.5 [28.9 - 36.1], and 46.7 [42.6 - 50.8] respectively. As in Studies 1A-1B, the modal participant reported using none of the heuristics, with substantial variation. According to the model fits, the average probability that participants used each heuristic was 13.1 [9.04 - 17.2], 29 [25.7 - 32.2], 21.2 [16.9 - 25.4] respectively. The modal participant used none of the heuristics, and there was some variation (although less than in Study 1).

Finally, to better contextualize the results involving attribute weights, Fig. S2 shows the distribution of reported and fitted weights across participants for each attribute (for Studies 1A and 2).

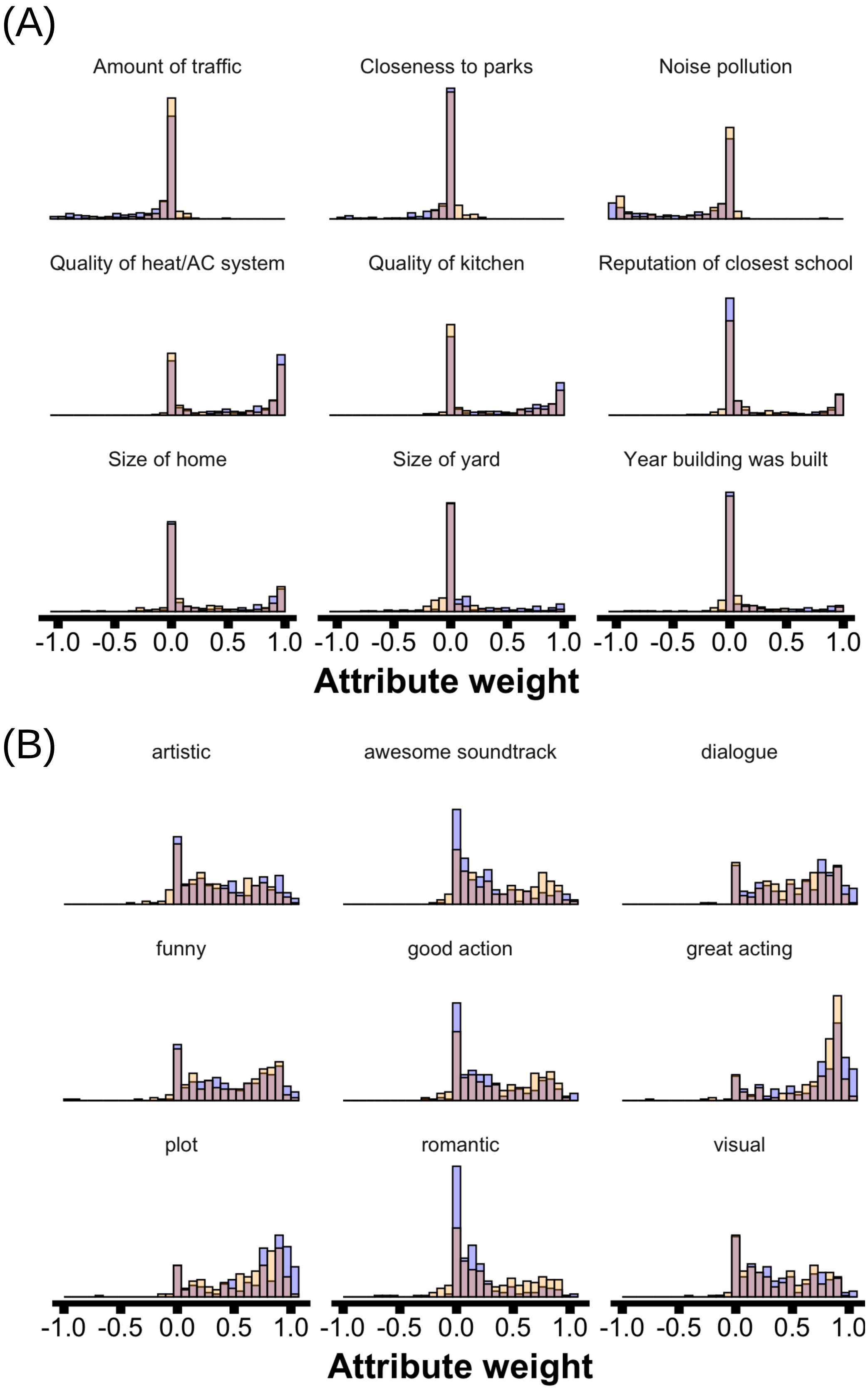
(A)
Amount of traffic
Closeness to parks
Noise pollution
Quality of heat/AC system
Quality of kitchen
Reputation of closest school
Size of home
Size of yard
Year building was built
-1.0 -0.5 0.0 0.5 1.0
Attribute weight
(B)
artistic
awesome soundtrack
dialogue
funny
good action
great acting
plot
romantic
visual
-1.0 -0.5 0.0 0.5 1.0
Attribute weight

Figure S2: Distributions of reported (blue) and best-fit (beige) attribute weights for each attribute, in (A) Study 1A and (B) Study 2. Light maroon indicates the overlap.

3.5. Analyzing method accuracy for each heuristic separately

In the main text, we analyzed participants' method accuracy aggregating across all three heuristics. Here, we separately analyze each of the three heuristics in Table 1 of the main text, and show that the findings replicate for each heuristic independently. Table S4 reports the method accuracy statistics for each heuristic in each study; the p values in the "heuristic error" column are from a one-sample t-test comparing to the chance level determined via simulation (in the manner described above), and the p values in the "% of participants correctly reporting…" column are from a chi-squared proportion test comparing to the chance level of 50%. The only non-significant results are the heuristic error test for the "binary weights" heuristic in Study 1A ($p = 0.06$), and the correlation between reported and model-estimated use of the "binary weights" heuristic in Study 2 ($p = 0.10$).

**Heuristic**	**Study**	**Correlation between reported and model-estimated use**	**Heuristic error**	**% of participants correctly reporting whether they used heuristic or not**
Single attribute	1A	r = 0.54 [0.44 - 0.62], p < .001 *	0.32 [0.29 - 0.36], p < .001 *	0.68 [0.62 - 0.74], p < .001 *
Single attribute	1B	r = 0.57 [0.48 - 0.65], p < .001 *	0.29 [0.26 - 0.33], p < .001 *	0.73 [0.67 - 0.78], p < .001 *
Single attribute	2	r = 0.56 [0.46 - 0.64], p < .001 *	0.20 [0.17 - 0.23], p < .001 *	0.87 [0.83 - 0.92], p < .001 *
Binary weights	1A	r = 0.18 [0.051 - 0.30], p = .006 *	0.36 [0.32 - 0.40], p = .06	0.60 [0.54 - 0.67], p = 0.002 *
Binary weights	1B	r = 0.24 [0.12 - 0.35], p < .001 *	0.34 [0.30 - 0.38], p = .003 *	0.63 [0.58 - 0.69], p < .001 *
Binary weights	2	r = 0.096 [-0.032 - 0.22], p = .10	0.27 [0.24 - 0.31], p < .001 *	0.73 [0.68 - 0.79], p < .001 *
Binary attribute values	1A	r = 0.22 [0.10 - 0.34], p < .001 *	0.34 [0.30 - 0.37], p = .001 *	0.61 [0.55 - 0.67], p = .001 *
Binary attribute values	1B	r = 0.26 [0.14 - 0.37], p < .001 *	0.34 [0.31 - 0.38], p < .001 *	0.64 [0.58 - 0.70], p < .001 *

Binary attribute values	2	r = 0.2 [0.075 - 0.32], p = 0.002 *	0.37 [0.33 - 0.40], p < .001 *	0.73 [0.68 - 0.79], p < .001 *

Table S4: Method accuracy statistics for each heuristic separately.

3.6. Correlation between weight and method accuracy

As a supplementary analysis, we examined the relationship between weight and method accuracy (while controlling for the confounds described in the next section). In all studies, participants' weight accuracy (i.e., the correlation between their reported and best-fit weights) was uncorrelated with their heuristic error (Study 1A: β = -0.03 [-0.17,0.11], t = -0.42, p = 0.68; Study 1B: β = -0.022 [-0.14,0.1], p = 0.72; Study 2: β = -0.11 [-0.24,0.017], p = 0.088). The results were similar for other measures of method accuracy (see online analysis code).

3.7. Confounders

We considered three potential confounders of accuracy: which choice method participants used, the quality of their model fit, and their comprehension of the self-report questions. For the purposes of this analysis, we operationalized these confounders as (respectively) the model-estimated probability that the participant used each of the three heuristics; the marginal likelihood of the participant's best-fitting model; and the number of comprehension check questions the participant got correct. The relationship between these confounding variables and accuracy scores is presented in Table S5 (weight accuracy) and Table S6 (heuristic error).

**Predictor**	**Study 1A**	**Study 1B**	**Study 2**
Model-estimated prob. of heuristic 1	β = 0.082 [-0.02,0.18], p = 0.11	β = 0.0064 [-0.051,0.064], p = 0.83	β = 0.35 [0.2,0.49], p < .001 *
Model-estimated prob. of heuristic 2	β = 0.021 [-0.11,0.15], p = 0.7	β = -0.066 [-0.14,0.011], p = 0.094	β = -0.35 [-0.53,-0.17], p < .001 *
Model-estimated prob. of heuristic 3	β = 0.15 [0.017,0.28], p = 0.027 *	β = -0.087 [-0.17,-0.0047], p = 0.038 *	β = 0.25 [0.12,0.39], p < .001 *
Marginal likelihood of best model	β = 0.03 [-0.49,0.55], p = 0.91	β = 1.0 [0.77,1.3], p < .001 *	β = 1.1 [0.43,1.7], p = 0.001 *
Correct # of CC questions	β = 0.015 [-0.044,0.074], p = 0.61	β = 0.019 [-0.019,0.056], p = 0.33	β = 0.067 [0.0061,0.13], p = 0.031 *

Table S5: Results when regressing weight accuracy on potential confounders. Asterisks indicate

significant relationships at $p < .05$.

Predictor	Study 1A	Study 1B	Study 2
Model-estimated prob. of heuristic 1	β = 0.17 [0.12,0.23], p < .001 *	β = 0.13 [0.084,0.17], p < .001 *	β = 0.064 [-0.0027,0.13], p = 0.06
Model-estimated prob. of heuristic 2	β = 0.087 [0.021,0.15], p = 0.01 *	β = 0.13 [0.074,0.19], p < .001 *	β = 0.066 [-0.016,0.15], p = 0.11
Model-estimated prob. of heuristic 3	β = -0.026 [-0.095,0.042], p = 0.45	β = -0.058 [-0.12,0.0028], p = 0.061	β = 0.0057 [-0.057,0.068], p = 0.86
Marginal likelihood of best model	β = -0.31 [-0.58,-0.034], p = 0.028 *	β = -0.38 [-0.59,-0.17], p < .001 *	β = -0.0034 [-0.3,0.3], p = 0.98
Correct # of CC questions	β = 0.0031 [-0.028,0.034], p = 0.84	β = 0.0037 [-0.024,0.032], p = 0.79	β = -0.013 [-0.041,0.015], p = 0.36

Table S6: Results when regressing heuristic error on potential confounders. Asterisks indicate significant relationships at $p < .05$.

3.8. Individual difference measures

We tested which of a battery of individual difference measures predicted accuracy. We grouped conceptually related measures into regressions together, regressing accuracy scores on them simultaneously. The groupings and results are reported in Table S7 (weight accuracy) and Table S8 (method accuracy). Each regression included the confounders from the last section as nuisance covariates. No measures in any study significantly predicted accuracy after controlling for multiple comparisons, and none consistently predicted accuracy across studies even without controlling for multiple comparisons.

Measure	Grouping	Study 1A	Study 2
Decision styles scale	1	β = -0.025, p = .736	β = 0.060, p = 0.36
Cognitive Affective Mindfulness Scale – Revised		β = .015, p = .88	β = 0.19, p = 0.039

Self-reflection and insight scale		β = .056, p = 0.47	β = -0.0016, p = 0.98
Multidimensional assessment of interoceptive awareness		β = -0.0087, p = .91	β = -0.096, p = 0.21
Attentional control scale		β = .054, p = .57	β = -0.077, p = 0.37
International cognitive ability resource		β = .11, p = 0.12	β = 0.13, p = 0.035
Gender (female > male)	2	β = -0.20, p = 0.15	β = -0.0098, p = 0.94
Gender (other > male)		β = 0.47, p = 0.35	β = -0.15 , p = 0.62
Age		β = 0.071, p = 0.33	β = 0.12, p = 0.048
Race (person of color > white)		β = 0.20, p = 0.20	β = -0.13, p = 0.39
Education level		β = 0.070, p = 0.34	β = 0.14, p = 0.026
Income level		β = 0.038, p = 0.61	β = -0.0098, p = 0.88
Native English speaker (yes > no)		β = 0.046, p = 0.89	β = -0.32, p = 0.51
Meditation experience (yes > no)	3	β = 0.20, p = 0.12	β = -0.076, p = 0.50
Alerting score from attention network test (ANT)	4	β = -0.066, p = 0.34	β = 0.023, p = 0.70
Orienting score from ANT		β = 0.15, p = 0.041	β = 0.081, p = 0.20
Executive score from ANT		β = 0.032, p = 0.64	β = -0.039, p = 0.51
Experience with choice domain	5	β = 0.023, p = 0.73	β = -0.025, p = 0.66

Experience with multi-attribute choice studies		β = 0.034, p = 0.61	β = -0.073, p = 0.21
Confidence in accuracy of self-reports	6	β = 0.051, p = 0.51	β = 0.064, p = 0.33
Satisfaction with choices		β = -0.042, p = 0.57	β = -0.057, p = 0.39
Self-reported attention paid	7	β = 0.015, p = 0.82	β = 0.040, p = 0.48

Table S7: Results from regressing weight accuracy on individual difference measures. See Table 1 above for measure descriptions & citations. Measures were grouped together in simultaneous regressions as shown in the "Grouping" column, although the results were all qualitatively similar when not grouping measures together. For simplicity, we omit Study 1B (the results are qualitatively similar to Study 1A), and only present standardized coefficients and p values; detailed results for all studies are available via the online analysis code. Cutoff for significance after Bonferonni correction was 0.05 / 24 = 0.0021.

**Measure**	**Grouping**	**Study 1A**	**Study 2**
Decision styles scale	1	β = 0.043, p = .56	β = 0.076, p = 0.32
Cognitive Affective Mindfulness Scale – Revised		β = .0056, p = .96	β = -0.21, p = 0.05
Self-reflection and insight scale		β = .054, p = 0.48	β = -0.00083, p = .99
Multidimensional assessment of interoceptive awareness		β = -0.0062, p = .99	β = 0.095, p = 0.29
Attentional control scale		β = -0.015, p = .12	β = 0.034, p = 0.74
International cognitive ability resource		β = .04, p = 0.57	β = -0.13, p = 0.074
Gender (female > male)	2	β = 0.93, p = 0.048	β = -0.053, p = 0.71
Gender (other > male)		β = 0.16, p = 0.21	β = -0.15, p = 0.67

Age		β = 0.033, p = 0.63	β = -0.16, p = 0.036
Race (person of color > white)		β = 0.27, p = 0.066	β = 0.19, p = 0.27
Education level		β = 0.0098, p = 0.89	β = -0.20, p = 0.0097
Income level		β = 0.0097, p = 0.89	β = 0.11, p = 0.16
Native English speaker (yes > no)		β = 0.41, p = 0.19	β = 0.063, p = 0.91
Meditation experience (yes > no)	3	β = -0.020, p = 0.87	β = -0.18, p = 0.16
Alerting score from attention network test (ANT)	4	β = 0.013, p = 0.85	β = 0.032, p = 0.66
Orienting score from ANT		β = 0.028, p = 0.68	β = 0.10, p = 0.16
Executive score from ANT		β = 0.049, p = 0.46	β = -0.12, p = 0.068
Experience with choice domain	5	β = 0.035, p = 0.57	β = -0.011, p = 0.87
Experience with multi-attribute choice studies		β = -0.065, p = 0.30	β = 0.011, p = 0.87
Confidence in accuracy of self-reports	6	β = 0.085, p = 0.23	β = 0.0022, p = 0.98
Satisfaction with choices		β = -0.10, p = 0.13	β = 0.052, p = 0.49
Self-reported attention paid	7	β = -0.059, p = 0.33	β = 0.012, p = 0.85

Table S8: Results from regressing heuristic error on individual difference measures. For simplicity, we omit Study 1B (the results are qualitatively similar to Study 1A), and only present standardized coefficients and p values; detailed results for all studies are available via the online analysis code. When interpreting these coefficients, keep in mind that lower heuristic error indicates greater accuracy. Cutoff for significance after Bonferonni correction was 0.05 / 24 = 0.0021.

3.9. Alternative approaches to analyzing attribute weights

In the main text, we report all weight accuracy results using weights averaged across the six choice models (weighted by the posterior probability of each model). But our key results do not depend on this analytic choice. For instance, if we focus on just the weights of the best-fitting choice model, the participant-level correlations between reported and best-fitting weights are 0.8 [0.75 - 0.84] (Study 1A), 0.85 [0.81 - 0.88] (Study 1B), and 0.59 [0.54 - 0.64] (Study 2). Alternatively, if we focus on just the weights of the reported choice model, the correlations are 0.71 [0.68 - 0.74] (Study 1A), 0.757 [0.733 - 0.781] (Study 1B), and 0.55 [0.50 - 0.59] (Study 2).

We also computed several alternative measures of weight accuracy that do not involve correlations. First, we computed the mutual information between each participant's reported and best-fit weights – equivalently, the amount of information gained about the best-fit weights by knowing the reported weights[37]. This measure is useful because it takes into account the amount of information available to learn in the best-fit weights; participants who accurately report using only one attribute (i.e., a simple distribution of weights) will score lower than participants who accurately report their graded weights across the full spectrum. We computed the mutual information using the *mi.empirical* function from the "entropy" R package[38], using the *discretize2d* function to discretize the weights into 10 bins. Participants' reported weights on average contained 0.91 [0.83 - 0.99] bits of information about their best-fit weights in Study 1A, 1.1 [0.98 - 1.1] bits in Study 1B, and 1.4 [1.4 -1.5] bits in Study 2 (all amounts significantly greater than zero, p's < .001). One way to contextualize this number is to compare it to the amount of total information that *could* be gained in principle about the best-fit weights (i.e., the amount of Shannon entropy contained in the best-fit weights). Participants' reported weights contained, on average, 79% [75% - 82%] of the total information that could be revealed about the best-fit weights (Study 1A; Study 1B: 81% [78% - 84%]; Study 2: 72% [70% - 74%]). Finally, and importantly, observers' reports consistently contained less information than deciders' reports (Study 3A: $\beta = -0.11$ [-0.03, -0.19], $t(180) = -2.7$, $p = .0071$; Study 3B: $\beta = -0.16$ [-0.05, -0.27], $t(176) = -2.9$, $p = .0046$).

To further probe the information revealed in participants' reported weights, we split the information gain metric by the participant's best-fit model, and compared it to the maximum amount of information that could be gained about the fitted weights in each case (Fig. S3). Fig. S3 shows the results for Study 1A; the other studies show very similar results. Participants who used more complex choice methods (such as the classically rational model and binary attributes model, which both use fully graded weights) revealed more information about their fitted weights from their reported weights, compared to participants who used simpler choice methods (such as choosing based on one attribute). On the other hand, they revealed a lower percentage of the total possible information. Across studies, participants who used graded weights (the left two models) reported weights that revealed about 70-75% of the information in the fitted weights; participants who used binary weights (middle two models) revealed about 60-80% of the information; and participants who used single-attribute weights (right two models) revealed about 85-95% of the information.

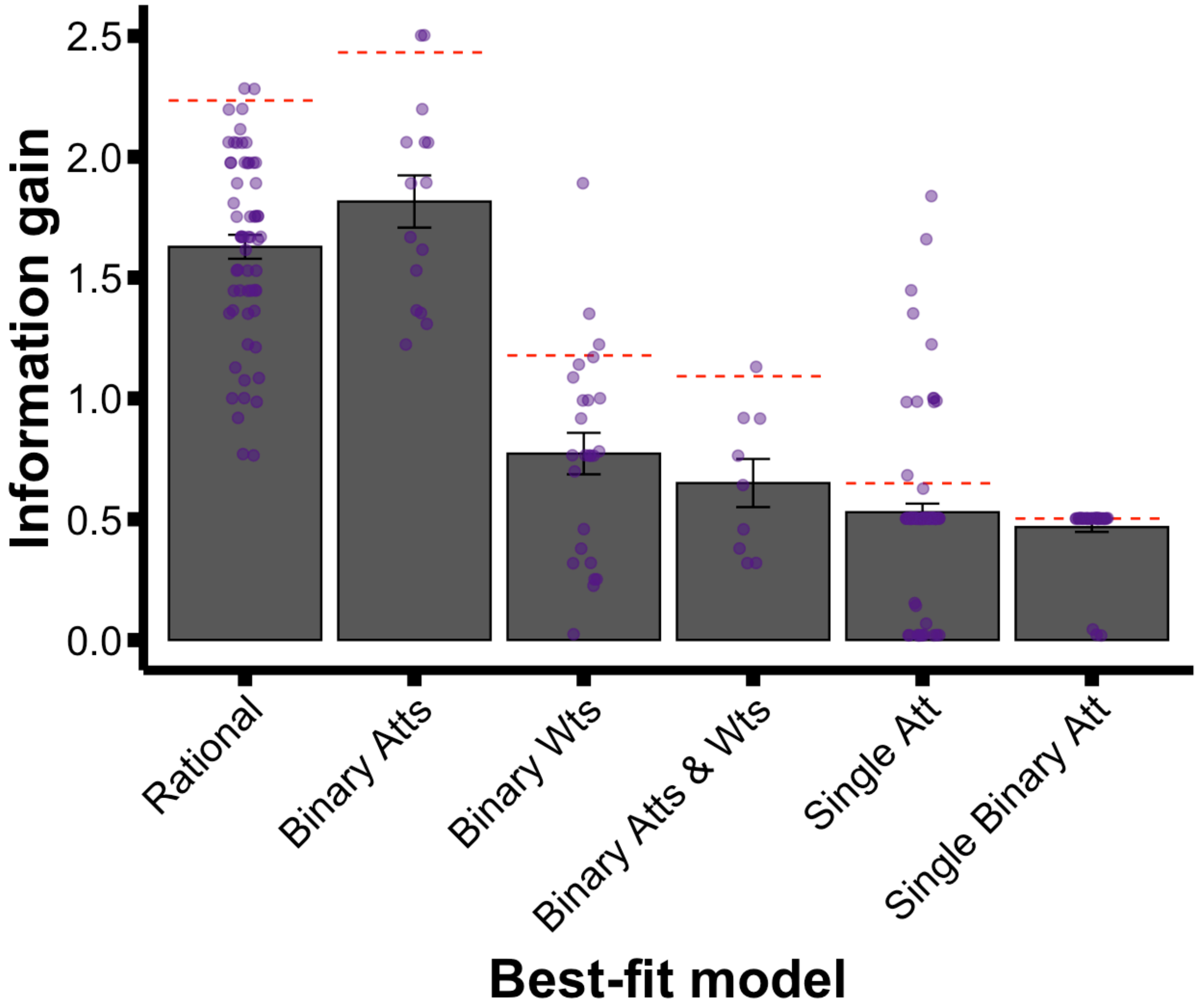

Figure S3: Bars indicate the amount of information gained (in bits) about fitted weights from reported weights, averaged across participants, split by best-fitting model in Study 1A. Red dashed lines indicate the maximum amount of information that *could* be gained about the fitted weights, again averaged across participants and split by best-fitting model. Purple dots indicate information gained by individual participants for each best-fitting model. Error bars indicate SEM.

As a final alternative measure of weight accuracy, we compute for each participant the absolute difference in magnitude between each of their reported and best-fit weights, and average across the nine attributes to obtain the participant's "absolute weight error". We find that participants exhibit relatively low levels of weight error (Study 1A: 0.13 [0.12 - 0.15]; Study 1B: 0.12 [0.11 - 0.14]; Study 2: 0.21 [0.20 - 0.23]; greater than chance, all p's < .001). As the weights ranged from -1 to 1, participants were generally accurate to within 1 part in 10 to 1 part in 20. We computed chance levels of weight error via a permutation test, and found that participants exhibited far less error than chance (all chance error levels around 0.35; all p's < .001). In addition, observers' reported weights exhibited significantly more error (Study 1A: $\beta = 0.34$ [0.20, 0.48], $t(211) = 4.7$, $p < .001$; Study 2: $\beta = 0.56$ [0.41, 0.71], $t(202) = 7.2$, $p < .001$).

In sum, participants exhibited high weight accuracy regardless of analytic method.

3.10. Comparison of weight accuracy results to those reported in past work

To get an approximate sense of how the weight accuracy found in the present studies compares to that found in previous work, we aggregated the prominent past studies we know of that measured weight accuracy in value-guided multi-attribute choice and included an observer condition. Taking a naive average across these studies, the average decider correlation between fitted and reported weights was 0.47; the average observer correlation between fitted and reported weights was 0.39; and thus the average decider advantage over observers was 25%. In contrast, we find decider correlations between 0.57 - 0.86 and decider advantages of 27-78%. Thus, we on average found higher weight accuracy than most past work, and the highest levels of weight accuracy we observe are higher than any found in past work.

**Paper**	**Decider *r***	**Observer *r***	**% decider advantage**
Nisbett & Bellows (1977)[20]	0.39	0.45	-13.3%
Wright & Rip (1981, study 1)[11]	0.38	0.32	18.8%
Wright & Rip (1981, study 2)[11]	0.58	0.4	45.0%
Kraut & Lewis (1982)[16]	0.42	0.35	20.0%
Wilson, Laser & Stone (1982)[23]	0.42	0.45	-6.70%
Gavanski & Hoffman (1986)[18]	0.41	0.23	78.3%
Gavanski & Hoffman (1987)[14]	0.71	0.53	34.00%

Table S9: Summary of weight accuracy scores (correlations between reported and fitted weights) found in past studies. The values for the first five are taken from the review in Wilson & Stone[24]; we added the Gavanski & Hoffman studies (for the 1987 study, we took the average of the correlation found in the three conditions).

4. Supplementary results for Studies 3a-3b

4.1. Are observers more accurate when they are demographically similar to their paired decider?

As a supplementary analysis, we tested whether the similarity between observers and their paired decider predicted observers' accuracy scores. We operationalized similarity with the five demographic measures we collected: gender, race, age, education level, and income (see SI 1.3 for exact questions). We numericized education level and income (such that the lowest level on each scale had a value of 1, the second had a value of 2, and so on). Then, for each observer, we computed a dissimilarity score between each of their demographic responses and those of their paired decider. For age, education level, and income, the score was simply the absolute difference between the two responses. For gender and race, the score was 1 if the observer's gender or race were different from the decider's, and 0 if they were the same.

Finally, we estimated linear regressions with the five dissimilarity scores as predictors and (separately) weight and method accuracy as dependent variables. (For our measure of method accuracy, we used 1 minus heuristic error scores.) A significant negative coefficient would indicate that more similar observers are more accurate. Bayes factors for the null hypothesis (i.e., that each coefficient was equal to zero) were computed using the BRMS package in R[39]; we re-computed the model with Bayesian regression, then estimated a null model with the target coefficient removed, and finally used the "bayes_factor" function to estimate the evidence in favor of the null model.

**Demographic dissimilarity**	**Study 1A: Weight accuracy**	**Study 1A: Method accuracy**	**Study 2: Weight accuracy**	**Study 2: Method accuracy**
Gender	β = -0.023, p = 0.73, BF_{null} = 5.8	β = -0.028, p = 0.32, BF_{null} = 8.6	β = -0.034, p = 0.58, BF_{null} = 5.6	β = 0.0038, p = 0.87, BF_{null} = 48.0
Race	β = -0.022, p = 0.73, BF_{null} = 6.0	β = 0.022, p = 0.41, BF_{null} = 10.3	β = 0.063, p = 0.30, BF_{null} = 3.8	β = -0.00048, p = 0.98, BF_{null} = 17.7
Age	β = -0.034, p = 0.27, BF_{null} = 7.0	β = 0.014, p = 0.32, BF_{null} = 19.6	β = -0.019, p = 0.53, BF_{null} = 11.0	β = -0.0054, p = 0.63, BF_{null} = 31.2
Education level	β = -0.017, p = 0.58, BF_{null} = 11.1	β = 0.0089, p = 0.50, BF_{null} = 23.8	β = 0.014, p = 0.66, BF_{null} = 11.6	β = 0.00025, p = 0.98, BF_{null} = 34.2
Income	β = -0.018, p =	β = 0.0037, p =	β = 0.0056, p =	β = 0.0012, p =

	0.59, BF_{null} = 10.5	0.79, BF_{null} = 27.0	0.86, BF_{null} = 12.6	0.92, BF_{null} = 34.3
Overall R^2	0.012	0.021	0.012	0.002

Table S10: Relationship between observers' accuracy and observers' demographic dissimilarity to their matched decider.

4.2. Variance decomposition of decider vs. observer reports

We analyzed the percentage of variance explained by observer reports, in two ways. First, we analyzed what percent of the variance in deciders' reports was explained by observers' reports. We fit a mixed-effects model regressing deciders' reports (separately, weights and heuristics) on their matched observer reports (with random effects for subject and item), and used the *r2beta* function (with method = 'nsj' and partial = TRUE) from the *r2glmm* package to quantify the percent variance explained by the observer report fixed effect[40]. We found that 17% [14% - 20%] (in Study 3A) and 4.4% [2.6% - 6.5%] (in Study 3B) of the variance in deciders' weight reports was explained by observers' weight reports, and 1% [0% - 3.7%] (Study 3A) and 0.7% [0% - 2.8%] (Study 3B) of the variance in deciders' heuristic reports was explained by observers' heuristic reports.

Second, we analyzed what percent of the variance in the fitted results were explained by deciders' vs. observers' reports. To do this, we fit a mixed-effect model regressing the best-fit results (separately, weights and heuristics) on the corresponding decider and matched observer reports, again with random effects for subject and item. Using the same *r2beta* function as above, we estimated the variance explained uniquely by deciders' reports; uniquely by observers' reports; and by their overlap. The results are shown in Table S10.

	% variance uniquely explained by deciders' reports	% variance uniquely explained by observers' reports	% variance explained by overlap	Ratio of variance explained by deciders to variance explained by observers: $\frac{Column\ 1}{Column\ 2\ +\ Column\ 3}$
Study 3A: Weights	39% [36% - 43%]	3.6% [2% - 5.7%]	15%	2
Study 3A: Heuristics	8.6% [4.6% - 14%]	2.8% [0.7% - 6.3%]	1.4%	2

Study 3B: Weights	30% [24% - 32%]	1.4% [0.5% - 2.8%]	4.4%	5
Study 3B: Heuristics	8.2% [4.3% - 13%]	0.7% [0% - 2.8%]	0.9%	5

Table S11: Percent of variance in best-fit weights and heuristics explained by deciders' vs. observers' reports. (Note that the *r2beta* function does not provide 95% confidence intervals for the percent variance explained by the variable overlap, and hence we do not report them here.) To conservatively compute the ratio of variance explained by deciders to the variance explained by observers, we divided the % variance uniquely explained by deciders (Column 1) by the sum of the variance uniquely explained by observers (Column 2) and the variance explained by their overlap (Column 3).

5. Supplementary results for Study 4

We compared experts' predictions to the actual average weight correlation (for weight accuracy) or percent of participants reporting all three heuristics correctly (for method accuracy) with one-sample t-tests. Experts predicted an average weight correlation of 0.44 (95% CI: [0.41 - 0.47]), which was significantly lower than the average correlation from Study 1A of 0.80 ($t(146) = -25.5$, $p < .001$) and from Study 1B of 0.86 ($t(146) = -30$, $p < .001$). It was similarly significantly lower than the average correlations after dropping participants who only used a single attribute (all p's < .001).

Finally, we tested whether experts' accuracy differed by role or field of study (Fig. S4). In separate regressions, we regressed the predicted weight and method accuracies on dummy variables indicating role and field of study (with "graduate student" and "judgment and decision-making" as the baseline values, respectively). After Bonferroni-correcting for multiple comparisons, the only significant effect was that full professors predicted a lower weight correlation ($b = -0.16$, corrected $p = 0.016$). The significance test for this difference reported in the main text is a two-sample t-test restricting to just graduate students vs. full professors.

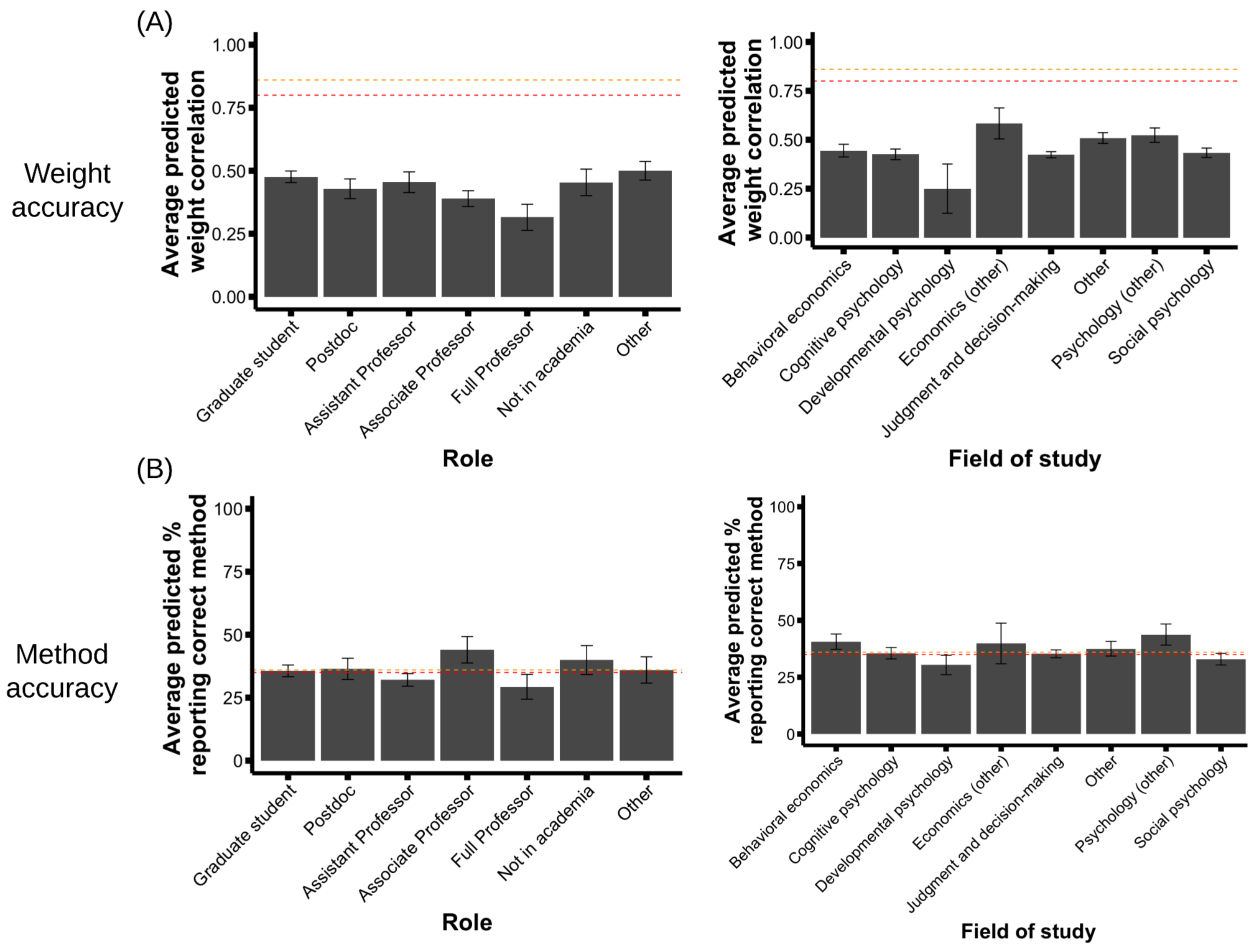

Figure S4: Experts' predictions about (a) weight accuracy and (b) method accuracy, split by role and field of study. Dashed horizontal lines indicate the average observed accuracies from Study 1A (red) and Study 1B (orange). Error bars indicate SEM.

Supplementary references

1. Harper, F. M. & Konstan, J. A. The MovieLens Datasets: History and Context. *ACM Trans. Interact. Intell. Syst.* **5**, 19:1-19:19 (2015).
2. Kotkov, D., Maslov, A. & Neovius, M. Revisiting the Tag Relevance Prediction Problem. in *Proceedings of the 44th International ACM SIGIR Conference on Research and Development in Information Retrieval* 1768–1772 (Association for Computing Machinery, New York, NY, USA, 2021). doi:10.1145/3404835.3463019.
3. Vig, J., Sen, S. & Riedl, J. The Tag Genome: Encoding Community Knowledge to Support Novel Interaction. *ACM Trans. Interact. Intell. Syst.* **2**, 13:1-13:44 (2012).
4. Fan, J., McCandliss, B. D., Sommer, T., Raz, A. & Posner, M. I. Testing the Efficiency and Independence of Attentional Networks. *Journal of Cognitive Neuroscience* **14**, 340–347 (2002).
5. Condon, D. M. & Revelle, W. The international cognitive ability resource: Development and initial validation of a public-domain measure. *Intelligence* **43**, 52–64 (2014).
6. Hamilton, K., Shih, S.-I. & Mohammed, S. The Development and Validation of the Rational and Intuitive Decision Styles Scale. *Journal of Personality Assessment* **98**, 523–535 (2016).
7. Derryberry, D. & Reed, M. A. Anxiety-related attentional biases and their regulation by attentional control. *Journal of Abnormal Psychology* **111**, 225–236 (2002).
8. Feldman, G. *et al.* Cognitive and Affective Mindfulness Scale-Revised (CAMS-R). in *Handbook of Assessment in Mindfulness Research* (eds. Medvedev, O. N., Krägeloh, C. U., Siegert, R. J. & Singh, N. N.) 1–24 (Springer International Publishing, Cham, 2022). doi:10.1007/978-3-030-77644-2_19-1.
9. Grant, A. M., Franklin, J. & Langford, P. The self-reflection and insight scale: A new measure of private self-consciousness. *Social Behavior and Personality: an international journal* **30**, 821–835 (2002).

10. Mehling, W. E., Acree, M., Stewart, A., Silas, J. & Jones, A. The Multidimensional Assessment of Interoceptive Awareness, Version 2 (MAIA-2). *PLOS ONE* **13**, e0208034 (2018).
11. Wright, P. & Rip, P. D. Retrospective reports on the causes of decisions. *Journal of Personality and Social Psychology* **40**, 601–614 (1981).
12. Cash, T. N. & Oppenheimer, D. M. Assessing metacognitive knowledge in subjective decisions: The knowledge of weights paradigm. *Thinking & Reasoning* **0**, 1–43 (2024).
13. Cook, R. L. & Stewart, T. R. A comparison of seven methods for obtaining subjective descriptions of judgmental policy. *Organizational Behavior and Human Performance* **13**, 31–45 (1975).
14. Gavanski, I. & Hoffman, C. Awareness of influences on one's own judgments: The roles of covariation detection and attention to the judgment process. *Journal of Personality and Social Psychology* **52**, 453–463 (1987).
15. Ikomi, P. A. & Guion, R. M. The Prediction of Judgment in Realistic Tasks: An Investigation of Self-Insight. *The International Journal of Aviation Psychology* **10**, 135–153 (2000).
16. Kraut, R. E. & Lewis, S. H. Person perception and self-awareness: Knowledge of influences on one's own judgments. *Journal of Personality and Social Psychology* **42**, 448–460 (1982).
17. Reilly, B. A. & Doherty, M. E. The assessment of self-insight in judgment policies. *Organizational Behavior and Human Decision Processes* **53**, 285–309 (1992).
18. Gavanski, I. & Hoffman, C. Assessing Influences on One's Own Judgments: Is There Greater Accuracy for Either Subjectively Important or Objectively Influential Variables? *Social Psychology Quarterly* **49**, 33–44 (1986).
19. Harries, C., Evans, J. S. B. T. & Dennis, I. Measuring doctors' self-insight into their treatment decisions. *Applied Cognitive Psychology* **14**, 455–477 (2000).
20. Nisbett, R. E. & Bellows, N. Verbal reports about causal influences on social judgments: Private access versus public theories. *Journal of Personality and Social Psychology* **35**,

613–624 (1977).

21. Nisbett, R. E. & Wilson, T. D. Telling more than we can know: Verbal reports on mental processes. *Psychological Review* **84**, 231–259 (1977).
22. Slovic, P. & Lichtenstein, S. Comparison of Bayesian and regression approaches to the study of information processing in judgment. *Organizational Behavior and Human Performance* **6**, 649–744 (1971).
23. Wilson, T. D., Laser, P. S. & Stone, J. I. Judging the predictors of one's own mood: Accuracy and the use of shared theories. *Journal of Experimental Social Psychology* **18**, 537–556 (1982).
24. Wilson, T. D. & Stone, J. Limitations of self-knowledge: More on telling more than we can know. *Review of personality and social psychology* **6**, 167--183 (1985).
25. Keeney, R. L., Raiffa, H., L, K. R. & Meyer, R. F. *Decisions with Multiple Objectives: Preferences and Value Trade-Offs*. (Cambridge University Press, 1993).
26. Payne, J. W., Payne, J. W., Bettman, J. R. & Johnson, E. J. *The Adaptive Decision Maker*. (Cambridge University Press, 1993).
27. Bhatia, S. & Stewart, N. Naturalistic multiattribute choice. *Cognition* **179**, 71–88 (2018).
28. Gelman, A., Lee, D. & Guo, J. Stan: A Probabilistic Programming Language for Bayesian Inference and Optimization. *Journal of Educational and Behavioral Statistics* **40**, 530–543 (2015).
29. Gronau, Q. F., Singmann, H. & Wagenmakers, E.-J. bridgesampling: An R Package for Estimating Normalizing Constants. *Journal of Statistical Software* **92**, 1–29 (2020).
30. Betancourt, M. A Conceptual Introduction to Hamiltonian Monte Carlo. Preprint at https://doi.org/10.48550/arXiv.1701.02434 (2018).
31. Vehtari, A., Gelman, A. & Gabry, J. Practical Bayesian model evaluation using leave-one-out cross-validation and WAIC. *Stat Comput* **27**, 1413–1432 (2017).
32. Penny, W. D. *et al.* Comparing Families of Dynamic Causal Models. *PLOS Computational*

Biology **6**, e1000709 (2010).

33. Bates, D., Kliegl, R., Vasishth, S. & Baayen, H. Parsimonious Mixed Models. *arXiv:1506.04967 [stat]* (2018).
34. Matuschek, H., Kliegl, R., Vasishth, S., Baayen, H. & Bates, D. Balancing Type I error and power in linear mixed models. *Journal of Memory and Language* **94**, 305–315 (2017).
35. Kuznetsova, A., Brockhoff, P. B. & Christensen, R. H. B. lmerTest Package: Tests in Linear Mixed Effects Models. *Journal of Statistical Software* **82**, 1–26 (2017).
36. Luke, S. G. Evaluating significance in linear mixed-effects models in R. *Behav Res* **49**, 1494–1502 (2017).
37. Latham, P. E. & Roudi, Y. Mutual information. *Scholarpedia* **4**, 1658 (2009).
38. Hausser, J. & Strimmer, K. Entropy Inference and the James-Stein Estimator, with Application to Nonlinear Gene Association Networks. *Journal of Machine Learning Research* **10**, 1469–1484 (2009).
39. Bürkner, P.-C. brms: An R Package for Bayesian Multilevel Models Using Stan. *Journal of Statistical Software* **80**, 1–28 (2017).
40. Jaeger, B. C., Edwards, L. J., Das, K. & Sen, P. K. An R2 statistic for fixed effects in the generalized linear mixed model. *Journal of Applied Statistics* **44**, 1086–1105 (2017).
