# Supplementary material for: Introspective access to value-based multi-attribute choice processes
Source: Nat Commun. 2025 Apr 20;16:3733. doi: 10.1038/s41467-025-59080-y (PMC12009298; doi:10.1038/s41467-025-59080-y)
Supplement: Supplementary file 2 — Reporting Summary [file 41467_2025_59080_MOESM2_ESM.pdf]

nature portfolio

Corresponding author(s): Adam Morris

Last updated by author(s): Jan 20, 2025

Reporting Summary

Nature Portfolio wishes to improve the reproducibility of the work that we publish. This form provides structure for consistency and transparency in reporting. For further information on Nature Portfolio policies, see our Editorial Policies and the Editorial Policy Checklist.

Statistics

For all statistical analyses, confirm that the following items are present in the figure legend, table legend, main text, or Methods section.

n/a	Confirmed	
☐	☒	The exact sample size (*n*) for each experimental group/condition, given as a discrete number and unit of measurement
☐	☒	A statement on whether measurements were taken from distinct samples or whether the same sample was measured repeatedly
☐	☒	The statistical test(s) used AND whether they are one- or two-sided<br>*Only common tests should be described solely by name; describe more complex techniques in the Methods section.*
☐	☒	A description of all covariates tested
☐	☒	A description of any assumptions or corrections, such as tests of normality and adjustment for multiple comparisons
☐	☒	A full description of the statistical parameters including central tendency (e.g. means) or other basic estimates (e.g. regression coefficient) AND variation (e.g. standard deviation) or associated estimates of uncertainty (e.g. confidence intervals)
☐	☒	For null hypothesis testing, the test statistic (e.g. *F*, *t*, *r*) with confidence intervals, effect sizes, degrees of freedom and *P* value noted<br>*Give P values as exact values whenever suitable.*
☒	☐	For Bayesian analysis, information on the choice of priors and Markov chain Monte Carlo settings
☐	☒	For hierarchical and complex designs, identification of the appropriate level for tests and full reporting of outcomes
☐	☒	Estimates of effect sizes (e.g. Cohen's *d*, Pearson's *r*), indicating how they were calculated

Our web collection on statistics for biologists contains articles on many of the points above.

Software and code

Policy information about availability of computer code

Data collection	Data were collected on Prolific; the experiment code was written in Javascript, using the jsPsych framework (version 6.3).
Data analysis	Data were analyzed in R version 4.3.1, with packages Stan (version 2.32.6, for model-fitting), and lme4 (version 1.1-34) and lmerTest (version 3.1-3) for mixed-effect model estimation. All code used in the manuscript can be found at https://osf.io/tmqju/.

For manuscripts utilizing custom algorithms or software that are central to the research but not yet described in published literature, software must be made available to editors and reviewers. We strongly encourage code deposition in a community repository (e.g. GitHub). See the Nature Portfolio guidelines for submitting code & software for further information.

Data

Policy information about availability of data

All manuscripts must include a data availability statement. This statement should provide the following information, where applicable:

- Accession codes, unique identifiers, or web links for publicly available datasets
- A description of any restrictions on data availability
- For clinical datasets or third party data, please ensure that the statement adheres to our policy

All data reported in this manuscript can be found at https://osf.io/tmqju/.

Research involving human participants, their data, or biological material

Policy information about studies with human participants or human data. See also policy information about sex, gender (identity/presentation), and sexual orientation and race, ethnicity and racism.

Reporting on sex and gender	We collected gender data (via self-report) from all participants for completeness, but did not analyze it separately as it was not relevant to any of our research questions. Gender was included as a potential individual difference covariate in the individual differences analyses reported in the main text; it had no significant effect in any of our studies. The source data can be analyzed split by gender via the analysis code provided at the OSF link.
Reporting on race, ethnicity, or other socially relevant groupings	We collected race/ethnicity data from all participants for completeness, but did not analyze it separately as it was not relevant to any of our research questions. Race was included as a potential individual difference covariate in the individual differences analyses reported in the main text; it had no significant effect in any of our studies. The source data can be analyzed split by race via the analysis code provided at the OSF link.
Population characteristics	See below.
Recruitment	For Studies 1-3, participants were recruited on Prolific and were nationally representative for age, race, and gender (in the U.S.). For Study 4, participants were recruited from the Society for Judgment and Decision-Making conference and associated email listserv. In Studies 1-3, participants were paid $12/hr for participation; in Study 4, participants did not receive compensation.
Ethics oversight	All participants gave informed consent. Princeton University Human Research Protection Program, Institutional Review Board; Yale University Human Research Protection Program, Institutional Review Board

Note that full information on the approval of the study protocol must also be provided in the manuscript.

Field-specific reporting

Please select the one below that is the best fit for your research. If you are not sure, read the appropriate sections before making your selection.

☐ Life sciences ☒ Behavioural & social sciences ☐ Ecological, evolutionary & environmental sciences

For a reference copy of the document with all sections, see nature.com/documents/nr-reporting-summary-flat.pdf

Behavioural & social sciences study design

All studies must disclose on these points even when the disclosure is negative.

Study description	Quantitative experimental
Research sample	Study 1A had 237 participants (53% female, 46% male, 1% other; mean age = 46 ± 16 years). Study 1B had 251 (53% female, 43% male, 4% other; mean age = 39 ± 14 years). Study 2 had 235 (50% female, 45% male, 5% other; mean age = 43 ± 15 years). Study 3A had 212 (50% female, 48% male, 2% other, mean age = 46 ± 16 years). Study 3B had 209 (58% female, 40% male, 2% other, mean age = 46 ± 16 years). The samples in Studies 1-3 were all collected on Prolific and were nationally representative for age, race, and gender (in the US). Recruiting participants on Prolific is an effective method for acquiring a reasonably representative sample (although participants may, of course, be non-representative along dimensions other than age, race, and gender). For Study 4, participants were recruited from the Society for Judgment and Decision-Making conference and associated email listserv; it had 147 participants (42% female, 55% male, 3% other; mean age = 38 ± 12 years). We recruited this sample in Study 4 because we were seeking experts in Judgment and Decision-Making to predict the results of our other studies.
Sampling strategy	For Studies 1-3, we selected N = 300 based on prior pilot data (and the number of subjects required to use Prolific's national representative sample feature). These samples are only representative of the U.S. population along age, race, and gender, and are not necessarily representative along other dimensions or of populations in other countries. For Study 4, we recruited as many decision scientists as we could from SJDM. The SJDM sample was a convenience sample and is not necessarily representative of the entire population of decision scientists.
Data collection	Studies 1-3 were conducted online via Prolific. Study 4 was run online, and participants were recruited from the Society for Judgment and Decision-Making conference and associated email listserv. Nobody was present for any study besides the participant (and hence it was irrelevant if the researchers were blind to experimental condition / study hypothesis). All participants gave informed consent.
Timing	Studies 1-3 were run between 6/16/2022 and 11/17/2022. Study 4 was run between 11/8/2024 and 12/25/2024.
Data exclusions	Across Studies 1-3, we excluded participants who did not complete all choice trials; who spent an average of less than 2 seconds per instruction screen; who got fewer than 4/6 of the comprehension check questions correct; who reported incoherent attribute weight directions (see SI 1.2 for details); or who tabbed away from the study more than 20 times. In Studies 1-2, we excluded participants who chose the right or left option on more than 80% of choice trials; in Study 3, we instead excluded participants who selected the correct button (i.e., the button highlighted as the original decider's choice) on fewer than 95% of choice trials.<br><br>After exclusion, 237 participants remained for analysis in Study 1a, 251 in Study 1b, 235 in Study 2, 212 in Study 3A, and 209 in Study

3B. Note that 22 of these participants did not complete the second session in Study 1a, 45 in Study 1b, 15 in Study 2, and 34 in Study 3A (there was only one session for Study 3B); we include these participants in our main analyses, and only exclude them from the individual-difference analyses requiring data from the second session.

For Study 4, the expert prediction study, we recruited 170 participants from the SJDM annual conference and email listserv (see SI 3.14 for details). We excluded participants who reported having heard of our ACP task before, leaving 147 for analysis.

All exclusion criteria were chosen in advance.

Non-participation	As the study was run on Prolific, we do not know how many participants dropped out/declined participation.
Randomization	Participants were not randomly assigned to different groups (there were no between-subject manipulations).

Reporting for specific materials, systems and methods

We require information from authors about some types of materials, experimental systems and methods used in many studies. Here, indicate whether each material, system or method listed is relevant to your study. If you are not sure if a list item applies to your research, read the appropriate section before selecting a response.

Materials & experimental systems

n/a	Involved in the study
☒	☐ Antibodies
☒	☐ Eukaryotic cell lines
☒	☐ Palaeontology and archaeology
☒	☐ Animals and other organisms
☒	☐ Clinical data
☒	☐ Dual use research of concern
☒	☐ Plants

Methods

n/a	Involved in the study
☒	☐ ChIP-seq
☒	☐ Flow cytometry
☒	☐ MRI-based neuroimaging

Plants

Seed stocks	N/A
Novel plant genotypes	N/A
Authentication	N/A
